# Effects of Radiation Dose on Lubricants: A Review of Experimental Studies

**DOI:** 10.1021/acsami.4c21220

**Published:** 2025-02-27

**Authors:** Michal Macha, Dominika Senajova, Tim Giles, Marco Calviani, Sylvain Girard, Matteo Ferrari

**Affiliations:** † CERN, CH-1211 Geneva 23, Switzerland; ‡ Imperial College London, SW7 2AZ London, United Kingdom; ¶ Université Jean Monnet, Saint Etienne, CNRS, Institut d’optique Graduate School, Laboratoire Hubert Curien UMR 5516, F-42023 Saint-Etienne, France; § Institut Universitaire de France (IUF) Ministère de l’Enseignement Supérieur et de la Recherche 1 rue Descartes, 75005 Paris, France

**Keywords:** lubricant, oil, grease, polymer, nanomaterials, radiation, ionizing radiation, γ radiation, neutron radiation, tribology, friction

## Abstract

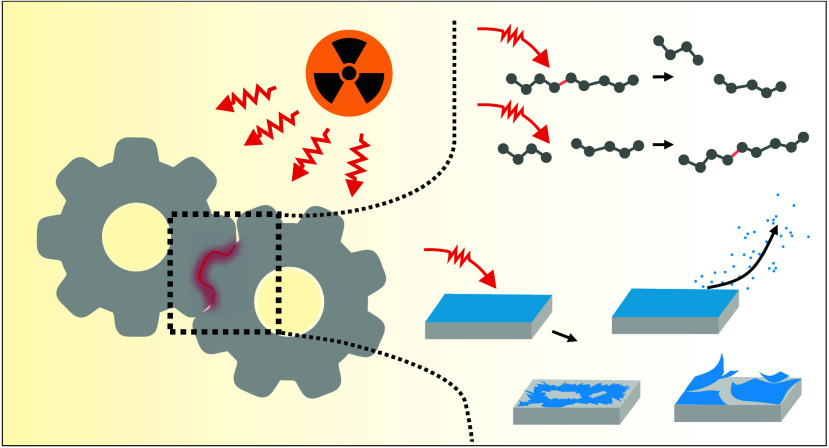

One of the key priorities in modern mechanical engineering is to ensure the safe, long-lasting operation of systems while maximizing energy efficiency and withstanding extreme and damaging conditions. This is especially relevant in environments subject to radiation, in which materials, depending on their chemical and elemental composition, exhibit specific limitations. In this review, we summarize the current state of the art in research and development regarding the performance of lubricants in radiation fields and the effectiveness of radiation-tolerant lubricated systems. We discuss the current understanding of radiation-damage mechanisms in dry-film lubricants, oils, and greases, and we summarize the experimental results of the irradiation studies performed to date. We compile and compare the established performance of various material types and chemistries in intense ionizing radiation fields. Finally, we provide an overview of future research directions, highlighting the challenges faced in irradiation studies, considerations relating to the selection of materials, new facilities and experiments aimed at advancing the field, and other issues related to the safe application and use of lubricant materials in radiation environments.

## 1 Introduction

One of the most important aspects of modern engineering domain is assuring safe, long-lasting operation of systems while maintaining their energy efficiency, avoiding damage, and protecting them from extreme working conditions. Energy losses in surfaces in mechanical contact are largely due to the presence and interaction of surface asperities. The resulting mechanical damage adds to surface degradation caused by reactive environments (such as those containing oxygen, water, salt, or corrosive chemicals), leading to a progressive decline in performance and potential failure. These technological problems can be addressed by applying lubricants—either in wet forms such as polymer-based oils and greases or as dry, paint-like coatings, such as graphite, molybdenum disulfide (MoS_2_), or polytetrafluoroethylene (PTFE, also known as Teflon). Coating surfaces with dry or wet lubricants allows for reduced wear, improved friction properties, and greater corrosion resistance, and it significantly minimizes the energy consumption of moving parts;^1,2^ however, lubricant materials are known to be much more susceptible to radiation damage than metals or ceramics.^3^ This represents a problem, especially in extreme engineering environments in which high radiation is coupled with high temperatures or oxygen exposure.

**Figure 1:**
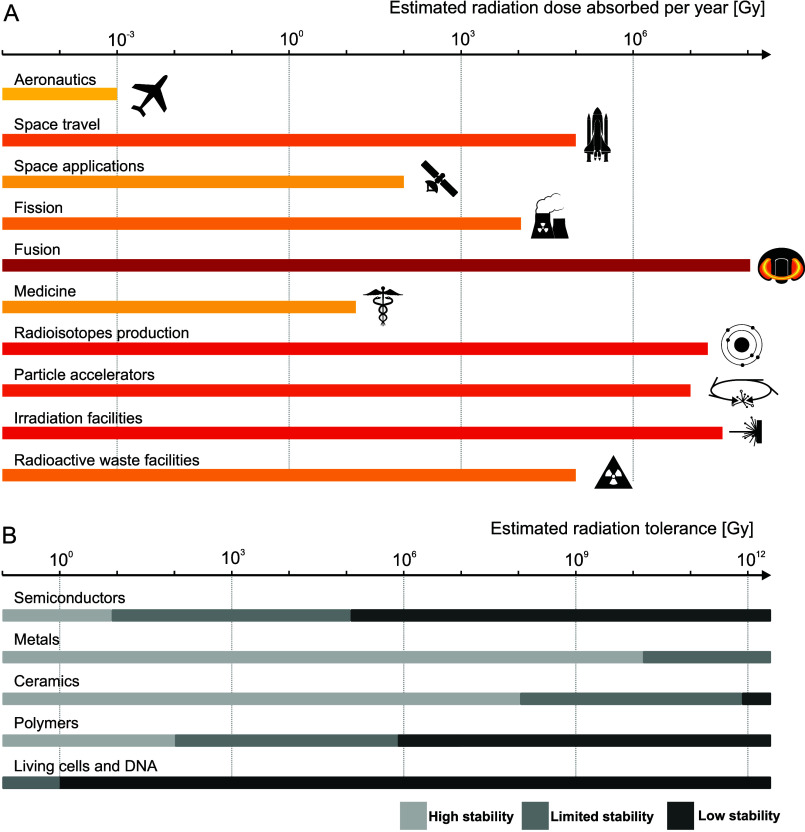
Classifications of (A) technological environments subjected to potentially damaging radiation and (B) radiation-tolerant materials,^48,49^ including estimated tolerance for DNA and living cells.^50,51^ Applications requiring radiation-tolerant materials and engineering solutions span diverse academic and industrial areas. The applications leading to the highest annual absorbed doses are fission,^17^ fusion,^11^ particle accelerators,^24,25,52^ radioisotope production,^30,53^ commercial irradiation facilities,^54^ radioactive-waste facilities and repositories,^55^ and space applications.^56,57^ Doses are expected to become more critical in future accelerator facilities.^28^ Presented estimated doses levels are based on the assumption of highest radiation dose that might influence mechanical moving parts rather than specific materials. Effective and safe operation in these fields is often limited by specific material categories and their intrinsic radiation-tolerance limits, leading to unique research, engineering, and maintenance challenges.^48,58^ In contrast to materials radiation tolerance, living cells undergo DNA damage with doses as low as few mGy^50^ with a lethal dose for humans estimated at 5–8 Gy for whole-body acute exposure.^59^

Despite the difficulties associated with lubrication in these environments, current knowledge in this field is mainly based on historical, unsystematic reports, anecdotal evidence, and rare, outdated research data. These specific application areas rely heavily on operation under significant absorbed radiation doses (Figure 1A). The estimation of the absorbed dose in lubricants as main reference quantity in this review is further discussed in Section 6.7. For example, in accelerator-driven applications, to minimize radiation damage, sensitive service materials such as lubricants are typically used as far as possible from radiation sources, including the primary beam (i.e., they are used off-beam). However, in many cases, space constraints or design considerations mean that lubricants must be used in equipment or locations where the radiation is the highest: these include interaction points where the particle beams collide, targets for production of secondary particle beams, collimators used to physically shape the circulating particle beams, and dumps designed to absorb the beams after use. There is therefore an urgent need to understand and develop novel radiation-resistant lubricants. This is particularly true given their use in industry and research sectors such as aeronautics,^4,5^ fusion,^6−15^ fission,^8,16,17^ space technology,^18−21^ present and future particle-accelerator technologies,^22−32^ nuclear medicine,^33−36^ and radioisotope production,^37−41^ which are experiencing a continuous and dynamic growth.

Given their criticality and ongoing technological development, reliable lubrication and surface protection will be needed for mechanical components that are exposed to increasingly higher doses. In addition, the dose levels typically encountered in operation (Figure 1A) might exceed the stability thresholds of conventional and commercially used radiation-tolerant lubricants. Due to the niche character and practical difficulties of research activities involving irradiation of materials, most studies were conducted between the 1950s and 1980s (see Section S1 of the Supporting Information). Only a few recent publications have reported the investigation of commercial products,^15,42−45^ various irradiation conditions, or the evolution of several lubricant properties,^21,42,45,46^ highlighting the need for current, state-of-the-art findings and the exploration of new and emerging materials.

Identifying and selecting lubricant materials with enhanced radiation resistance is crucial for minimizing the risks of failure and health impacts (such as the radiation dose to operators), reducing maintenance and repair costs, and mitigating environmental effects in both standard operations and accident scenarios.^47^ To this end, this paper aims to critically review, organize, and consolidate the existing literature and experimental findings on the application of lubricant materials in high-radiation environments with a view to providing a valuable reference for selecting suitable lubricants for these conditions.

The remainder of this review is structured as follows. Section 2 contains general definitions of the main types of materials that have been analyzed and the units and quantities considered in these analyses. Section 3 presents a summary of the fundamental knowledge about the effects of radiation on solid materials, polymers, and material interfaces in the context of wet and dry lubricants. A summary of the dry lubricants that have been investigated to date is provided in Section 4, including recent discoveries and studies on the application of nanomaterials in radiation environments. State-of-the-art knowledge about commercial radiation-resistant wet lubricants—specifically oils, greases, and lubricant additives—is presented in Section 5. The current research challenges related to performing reliable material studies and the existing state of knowledge are discussed in Section 6. The outlook and potential prospects for the field are presented in Section 7; this is followed by a summary of general conclusions and some closing remarks in Section 8.

## 2 Overview and Definitions

The primary function of lubricants is to reduce friction between moving parts in contact by forming a thin, low-shear layer between their surface asperities. This role is significantly harder to effectively fulfill in high-radiation environments, in which molecular and chemical damage occurring through irradiation can lead to a decrease in tribological properties. Fundamental aspects of lubricants, including their standard characteristics and measured properties—such as the coefficient of friction (CoF), total acid number (TAN), and viscosity—are outlined in Section S2 of the Supporting Information. Although an elemental understanding of lubricant properties is essential, it is equally important to understand the complexities of using these materials under specific irradiation conditions. An overview of these complexities is provided here.

### 2.1 Selection of Commercial Products

In a conventional environment, the choice of a wet or dry lubricant material and its specific chemistry should ideally be dictated by the machine function, desired tribological properties (such as CoF or wear resistance), surface chemistry (chemical affinity for or inertness to the chosen lubricant), contact pressure, and working atmosphere. In real-life scenarios, however, lubricant products are often selected based on their general properties, such as worked consistency for greases and kinetic viscosity for fluid oils. These quantities can be easily and routinely measured via standard tests, and their values are normally reported on product datasheets. While this approach lacks specificity, it allows designers and equipment owners to choose commercially available lubricants.

Exposure to ionizing radiation and susceptibility to radiation damage add another layer of complexity. Criteria for the selection and testing of commercial lubricants for high-radiation applications will be further discussed in Section 7.

Due to the complexity of the chemical compositions of lubricants and the processes involved in radiation-induced molecular damage, the efficiency and performance of a lubricant often degrade—sometimes nontrivially—in radiation environments.^60−62^ Considering radiation resistance is therefore of paramount importance in the design of materials systems (Figure 1B) when seeking to maximize their lifetime, performance, and safety factors.^63^

### 2.2 Material Interfaces

Lubricants, whether wet or dry, are designed to be in contact with mechanical components (surfaces). After irradiation, it is imperative not only that each component retains optimal performance and properties but also that compatibility between the materials in contact is preserved. In practice, radiolysis products from one material can detrimentally impact the other material. For instance, phenomena such as hydrogen embrittlement^64^ and metal corrosion^42^ may result from contact between metallic substrates and hydrogen gas or acids produced within irradiated polymeric lubricants. This underscores the critical importance of evaluating and maintaining compatibility between materials that are exposed to radiation.

### 2.3 Radiation Environments

Ionizing radiation consists of high-energy particles that have sufficient energy to ionize atoms or molecules and, hence, deposit energy in matter. This radiation can be composed of electromagnetic waves (such as X-rays and γ rays) or subatomic particles (such as protons, neutrons, electrons, pions, kaons, alpha particles, and heavy ions) with energies exceeding the ionization threshold, conventionally set at around 10 eV. When more than one radiation species is present in a radiation field, it is referred to as a “mixed” field.

In most materials radiation studies, the total accumulated physical absorbed dose (i.e., energy per unit mass expressed in Gy (=J/kg) or equivalent units, such as the rad)^65^ is used as the quantity to which the effects, damage levels, and thresholds of radiation are referred.^3,11,15,42,43,49,54,60,66−75^ The total integrated fluence and particle fluxes are sometimes specified in cases of mixed-field and neutron irradiation.^13,15,42,52,75,76^ As will be discussed in Section 6.4, the effects of radiation are expected to depend not only on the total absorbed dose but also a wide array of other irradiation parameters such as the radiation type and energy spectrum, dose rate, atmosphere, and temperature. Nonetheless, due to the lack of systematic parametric investigations, and taking a pragmatic approach, we have chosen in the present paper to adopt the total dose as a primary reference parameter to compare the analyzed studies, while recognizing that this represents an oversimplification of the actual scenario. The limits of this approach are discussed further in Section 6.7.

### 2.4 Radiation Resistance

The concept of *radiation resistance* lacks a consistent definition in literature. Usually, authors refer to materials as *radiation resistant* if a selected material property remains stable after irradiation up to a certain level within a given percentage, or if it simply seems to remain unchanged. However, the properties selected to assess stability among those tested, the irradiation conditions, the test method, and the acceptable variation vary significantly between authors. For example, Beynel et al.^49^ consider a 50% change in the most sensitive among the tested property of a given material or component as usable end point to mark a dose threshold for radiation tolerance.

Various authors agree on the use of variations in kinetic viscosity as the most relevant quantity for fluid oils, and many consider relative variations of 20%^43,72,77^ or of 100%^75,78^ at 40 °C as generic thresholds.

Concerning greases, changes in worked consistency, as measured by cone penetration, are mostly used as a threshold. Nonetheless, there is again no consensus on the relative variation to be used, with values typically in the range 10%—-15%.^12,42,73,74,79^

We can simplify the definition of *radiation resistance* as the “stability of a selected property within a defined range of values that is compatible with a generally agreed-upon standard or specific operational requirements”, although it is noted that this definition still lacks general agreement on the tested property and on the allowed variation range. In this context, stability can be achieved in several ways, depending on the specific environmental and operational conditions and material and system properties (see Section 6.4), and it requires a broad analysis of the engineering problem in the context of the desired machine function. To this end, it is important to understand the mechanisms by which radiation causes damage to lubricating materials.

## 3 Radiation-Damage Mechanisms

Typical applications analyzed in experimental studies showcase work environments reaching the MGy range of absorbed doses. At this level of radiation, material damage is nontrivial, is often very complex, and can have a dramatic impact on the macroscopic properties of materials and operational safety. It is, therefore, crucial to understand the most relevant mechanisms occurring in both the crystalline materials used in various dry lubricants and organic polymers, which are the main components of oils and greases.

### 3.1 Solid Crystalline Materials

**Figure 2:**
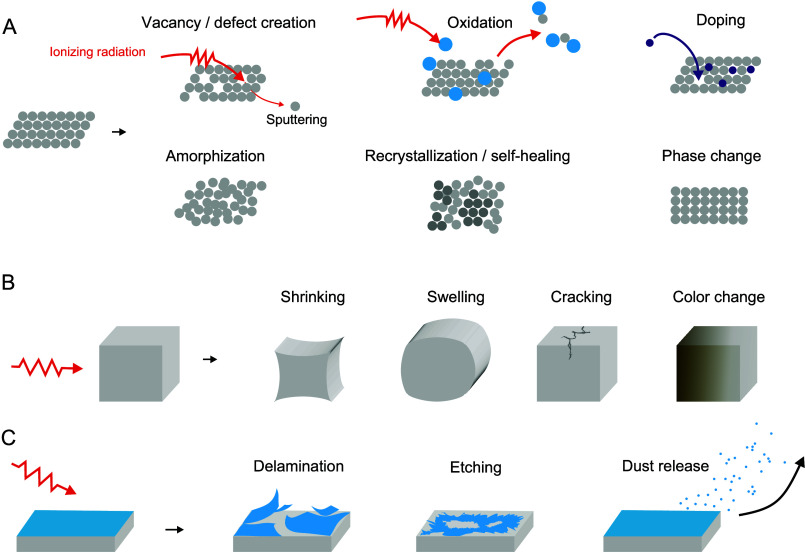
General effects of radiation damage. (A) Schematic representation of the most relevant radiation-damage phenomena occurring in materials at the atomic scale due to atomic displacement. (B) These changes lead directly to macroscopic changes in the bulk properties of a material. (C) Accumulation of radiation damage may lead to surface deterioration.

Radiation typically impacts the structure of a solid (Figure 2A), and this may lead to visible changes on the macroscale (Figure 2B,C). In crystalline materials in particular, the most relevant effect of radiation is atomic displacement caused by elastic and inelastic scattering processes triggered by radiation interacting with the bulk material.^80−82^ This ultimately results in the formation of interstitial vacancies and defects in the crystal lattice,^83^ which may induce local recrystallization (e.g., sp^2^ to sp^3^ transformation in the case of graphite) and, with increasing absorbed dose, a gradual amorphization, leading to a modification of the macroscopic properties of the material (e.g., dimensional changes, decreased mechanical and tribological properties, or etching).^84−87^ This recrystallization and the presence of local defects can, in some cases, be reversed by thermal annealing when triggered by a moderate increase in the radiation dose^86−91^ (discussed in more detail in Section 4). Typically, this effect is present until a critical dose is reached and atomic disruptions dominate, leading to progressive degradation of material properties. Overall, the atomic disruption (displacement damage), the rate of subsequent damage, and any potential self-annealing (i.e., thermal annealing) phenomena depend on the mass of the material, its density and crystal structure, and the energy and type of ionizing particles.^92,93^

Other phenomena that can have a severe effect on the practical use of a material and its application in a radiation environment are radiolytic oxidation and radiation-triggered chemical processes. Although neutron and γ radiation produce similar macroscopic effects, it is hypothesized that at equal doses, the former has a stronger effect on oxidation than the latter due to its higher rate of triggered atomic recoils and collision cascades.^83,94,95^ Atomic displacement and the formation of defect sites might lead to an enhanced rate of volatile-particle adsorption and oxidation. Under common irradiation conditions, oxygen is likely to be concentrated at the external surfaces, and this is directly influenced by the working environment to which the material is exposed (i.e., the oxygen content of the local atmosphere).

Aside from the direct effects on the materials, irradiated materials may themselves also impact surrounding elements by releasing radicals or sputtered particles. This effect can be observed on a macroscopic scale with sulfides (e.g., MoS_2_) which, under irradiation, may release sputtered sulfur atoms, potentially leading to stress corrosion of neighboring steel substrates.^96−98^ With composite materials—e.g., dry lubricating coatings—radiation may damage the composite binder, decreasing the structural stability of the coating. Such an irradiated film could release volatile, airborne, activated particles such as graphite.

All of these effects will directly impact the application of a material in a radiation environment and must be carefully considered and addressed to maintain operational safety.

### 3.2 Organic Polymers

Polymeric materials consist of large numbers of long molecular chains (macromolecules), originating from the repetition of base units.^99^ These molecular chains do not have a structure as ordered as the one of crystalline solids; they can display a multiplicity of possible arrangements and space orientations. Typically, chains can bend, coil, and be intertwined. Polymeric materials can have a certain degree of cross-linking, meaning that adjacent chains are linked to each other at one or multiple points via the nonreversible creation of covalent bonds. Cross-linking can be induced by specific reactions and/or by exposure to ionizing radiation. Overall, this leads to an irregular and amorphous material structure that, in some cases, shows a certain degree of crystallinity. The randomness of the molecular arrangement significantly impacts the main characteristics of a polymer and the overall mechanical performance of polymer-based materials.

In general, the properties of commercial polymer-based materials, including their tolerance to radiation, are critically dependent on the chemical nature of the base polymer and its molecular arrangement, the amount and chemistry of the additives (which are normally included to improve the performance of the material), the overall polymer structure (which is significantly influenced by the degree of cross-linking), and the entire process leading to the formulation of a specific material. These are the parameters taken into consideration in the present paper to analyze and classify the effects of radiation on liquid and semisolid polymer-based lubricants.

**Figure 3:**
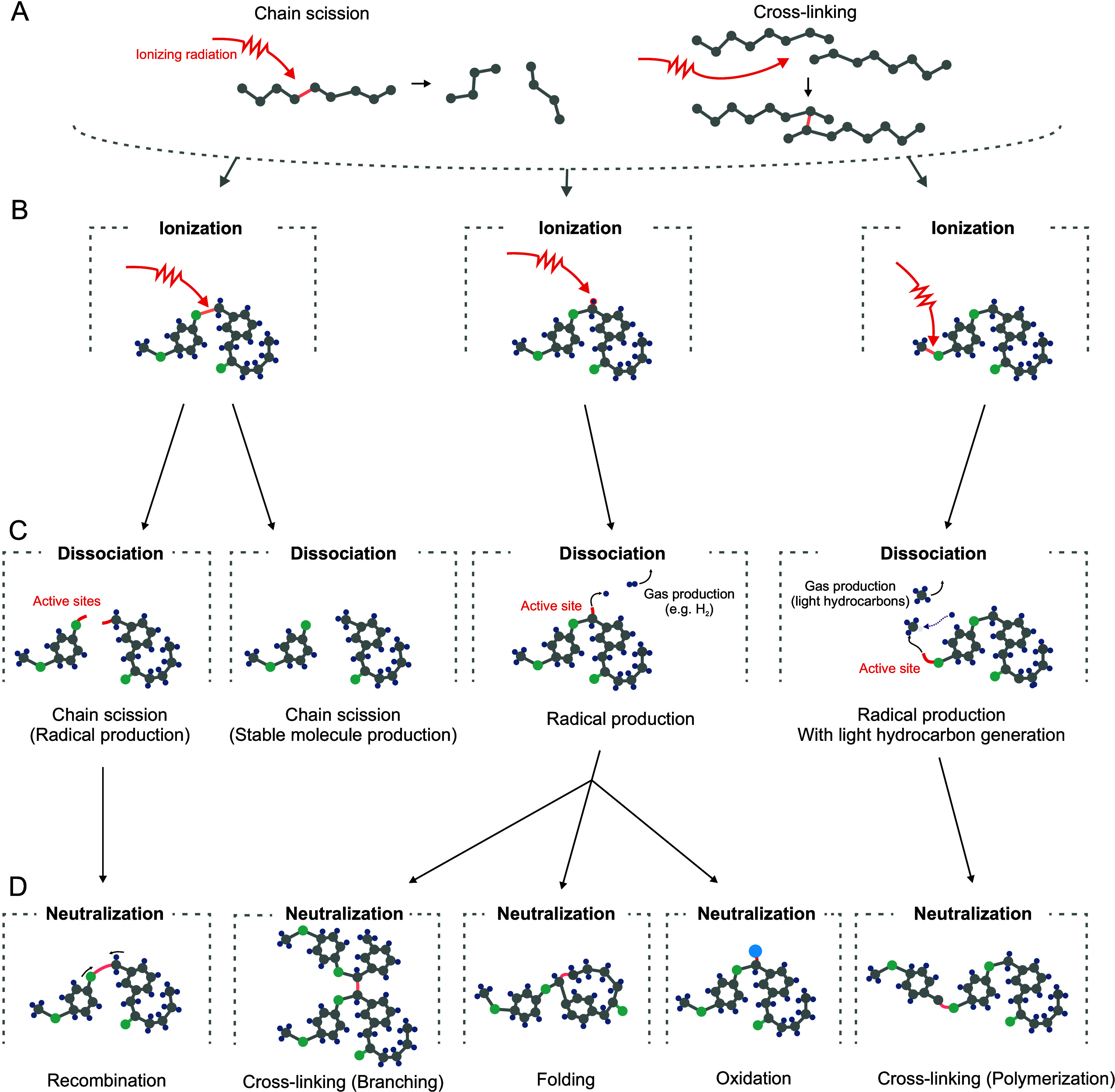
Effects of radiation damage on polymer molecules. (A) Polymer structures, in particular, undergo complex processes due to their molecular arrangements; these changes can be simplified to chain-scission and cross-linking phenomena. In a more detailed view, molecular damage can trigger the following process pathways: (B) ionization (excitation) by energetic particles, which, depending on the affected bond, lead to (C) dissociation processes of chain scission, radical, molecule, or gas production (e.g., light hydrocarbons), which may be followed by potential (D) neutralization processes of recombination, cross-linking, folding, and/or oxidation^3^

Polymers are generally more radiation sensitive than metals (Figure 1B)^3,48^ and, due to the diverse nature of organic chemistry and molecular arrangement, the effects of radiation on polymers are significantly more complex to define, categorize, model, and potentially anticipate.^68,100^ In polymer structures, the effects of radiation stem from physical and chemical processes induced by the total energy deposited by the interaction of radiation with the target material.^3^ Radiation transfers energy to atoms and molecules through a high number of possible interactions^65^ (see Figure 3). Ultimately, depending on the amount of energy transferred, radiation induces excitation and ionization, leading to molecular dissociation and possibly producing active sites, ions, and smaller molecules. In this sense, rather than the number of atomic displacements, it is more relevant to evaluate the energy deposited leading to excitations and ionizations; thus, the total absorbed dose becomes significant.

The effects of radiation on polymers include changes to molecular bonds, which break and recombine via the fundamental mechanisms of chain scission and cross-linking. These mechanisms lead to modifications in the weight or length of polymer chains (polymerization), changes in network structure and distribution, and the addition of molecular components (alkylation, oxidation) or their loss (desulphurization, decarboxylation, dehydrogenation).^3,43,48,49,66,68−70,75,101,102^

Free radicals can be created in these processes (see Figure 3), both in the irradiated material and the oxygen present in the atmosphere, and these can act as damage mediators. These radical species can either recombine or diffuse and interact with surrounding molecules in various reactions. Their accumulation, which is common in solid materials, is less relevant in semisolid materials (such as grease), in liquids, and in thin layers and films (e.g., lubricating films) due to their greater mobility.^66^

Some molecular bonds are more resistant to radiation-induced dissociation than others;^103^ for example, in aromatic rings, the energy is not absorbed by a single bond but is distributed throughout whole ring.^3^ This leads to a higher resistance to subsequent macroscopic effects. The same argument can be used to support investigating temperature-resistant lubricants as promising candidates for radiation applications.^78,103^ However, the example of fluorocarbons shows that thermal and radiation resistance are not always correlated.^3,42,104,105^

In polymers, several potentially competing mechanisms and effects can occur on both micro- and macroscopic scales, adding to the complexity of the damage mechanisms. The type and prevalence of the reactions occurring depend strongly on the polymer’s chemical composition and structure and the specific irradiation conditions. Temperature and environmental atmosphere (e.g., oxygen concentration), for example, can greatly affect the intensity, dominance, and even nature of the damage mechanisms.^3,66,70,100^

All these mechanisms play a role in determining the overall modifications of the material properties. Cross-linking and chain scission can occur simultaneously and concurrently;^75^ identifying whether one or the other prevails is relevant, as this will generally translate into increased or decreased material hardness.^42^ Macroscopically, radiation can affect the physical, structural, chemical, mechanical, electrical, and optical properties of polymers, including their density, color, glass transition temperature, melting point, elastic modulus, hardness, brittleness of solids, viscosity of liquids, etc.^3,49^ In some cases, radiation can induce the formation of volatile gaseous byproducts in quantities large enough to increase internal pressure and stresses. Outgassing can lead to work-environment contamination or equipment damage due to overpressure or chemical reactions.^42,64,69^

With such a wide variety of radiation-induced phenomena and the complex relationships between them, the assessment of the radiation tolerance of polymer materials for specific uses in extreme radiation environments is challenging; critically, it depends on the possibility of performing *ad hoc* irradiation tests (as further discussed in Section 7) and looking for correlations between micro- and macroscale effects.^63^

## 4 Dry Lubricants

**Figure 4:**
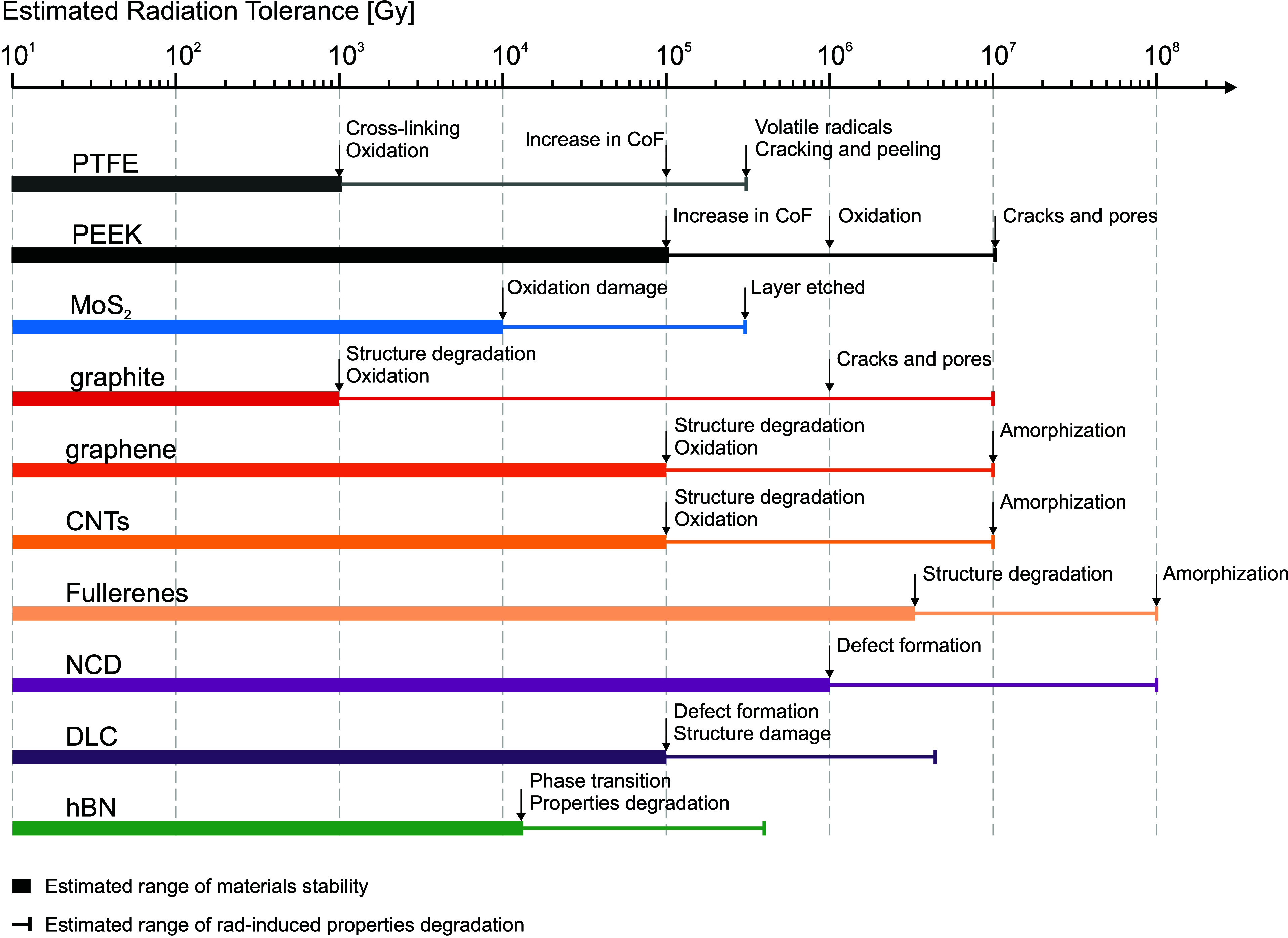
Estimated radiation-tolerance ranges of dry-film lubricants: PTFE,^110−114^ polyetheretherketone (PEEK),^115−118^ MoS_2_,^85,119−122^ graphite,^83,89,90,123−127^ graphene,^87,128,129^ carbon nanotubes (CNTs),^89,130−134^ fullerenes,^135−137^ nanocrystalline diamond (NCD),^138^ diamond-like carbon (DLC),^139^ hexagonal boron nitride (hBN),^140^ and examples of composite materials.^141−144^ The lines indicate reported or estimated range of radiation stability end points, indicating uncertainty regarding the usability of these materials in the specific radiation dose range due to a lack of specific radiation studies aimed at studying tribological properties.

Solid-state lubricants are, by design, suitable for use in conditions under which wet lubrication is limited. The most widely used of such materials demonstrate applicability in radiation conditions and have the potential for radiation tolerance; however, there have been few studies of their lubrication performance in high-radiation conditions (>1 MGy). In general, increasing the radiation dose will degrade the tribological properties of conventional solid lubricating materials, albeit at a slower rate than in organic-chemistry-based wet lubricants. As noted earlier, radiation induces disordering of the crystalline lattice, vacancy accumulation, lamellar voids in the case of layered materials, and amorphization. Initial studies of nanomaterial-based solid lubricants have shown significant improvements compared to conventional materials. Their possible applicability in high-radiation environments stems from the energy-dispersing character of nanoparticles and the high density of nanoscale grains, which can act as defect sinks.^106−109^ Further improvements in the radiation resistance of dry films can potentially be achieved by using nanocomposites, multilayer structures, and periodic thermal annealing to relax and stabilize defect phases. Nonetheless, solid-state, dry nanostructured and nanoparticle-infused lubricants represent an emerging field of research, and their applications are as yet limited by experimental constraints and small-scale synthesis methods. This is true, for example, in the case of potentially promising lubricating coatings based on fullerenes, nanotubes, or nanocomposite films. A summary of the estimated radiation-tolerance of the dry-film lubricant materials discussed in this section is presented in Figure 4.

### 4.1 Conventional Dry Lubricants

**Figure 5:**
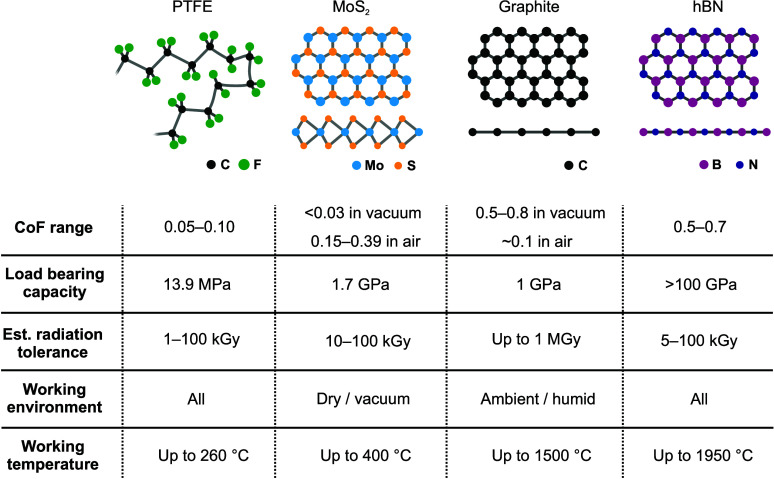
Comparison of the properties of the dry-film lubricants most frequently used in modern industry: polytetrafluoroethylene (PTFE),^112^ MoS_2_,^148^ graphite,^148,151,156^ and boron nitride (BN).^146^ Their specific application strictly depends on the working environment and load conditions of the machinery. Radiation-tolerance considerations add an additional level of complexity and introduce potential limitations when assessing the usability of a coating.

Dry-film lubricants are most commonly used in extreme applications such as high-temperature and high-vacuum situations where conventional organic materials (oils) fail to improve tribological properties or ensure surface protection.^145−150^ The variety of tribological properties achievable using dry lubricant materials makes them potentially attractive for use in high-radiation environments (i.e., >1 MGy annual dose; see Figure 5). In principle, a dry lubricant should form a stable film between sliding/rolling surfaces that is compatible with the specific working conditions. Most commonly used solid lubricant materials exhibit weakly bonded, lamellar crystal structures,^151,152^ the layers of which slip under low shear forces, enabling self-lubrication. As outlined in the previous section, in principle, the accumulation of radiation damage in solid materials leads to the loss of macroscopic properties, amorphization, and a potential increase in oxidation rates due to higher local reactivity; however, after absorption of specific dose levels in controlled irradiation conditions (i.e., below 1 MGy), some materials may exhibit *improved* tribological properties.^142^ To ensure a long lifetime, an ideal dry lubricant film should show stable surface adhesion and wear resistance or self-healing (i.e., defect-healing) properties.^148^ From an operational-safety perspective, to minimize potential radioactive contamination, the coated surfaces should not release any airborne dust during operation, should not form long-lived radioactive isotopes, and should not form or release any potentially toxic compounds.^153^ Finally, as with liquid lubricants, any radiation-triggered chemical changes in the lubricating film should not be damaging to the coated surface.^154^ These fundamental requirements limit the choice of potentially usable lubricants, excluding materials such as soft metals, sulfides, fluorides (e.g., PTFE), and most polymer-based coatings.^153,155^

#### PTFE

The intrinsic self-lubrication properties, chemical inertness, and thermal stability of PTFE mean that it is one of the most commonly used dry lubricant materials.^112^ Unfortunately, due to its extreme sensitivity to radiation-induced damage, it has limited use in radiation applications. When PTFE is exposed to radiation, chain scission and cross-linking are the predominant effects, leading to an increase of the CoF and a deterioration of mechanical properties manifested by material peeling and cracking. At an absorbed dose level of 0.3 MGy, the CoF increases from 0.035 to 0.120; exposure to 5 MGy leads to a dramatic increase in wear rate, high-density surface cracking, and peeling.^112^ Although it is unsuitable for high-radiation environments, PTFE might be applicable below 150 kGy, primarily when used in composite coatings (e.g., with graphite^142^), mitigating its radiation-induced deterioration.

#### MoS_2_

Molybdenum disulfide (MoS_2_) is one of the most commonly exploited solid lubricants, and it is widely used in vacuum applications. It is structurally similar to graphite or graphene, and its lamellar crystalline structure inherently allows for easy shear.^148^ However, the application of MoS_2_ in radiation fields is limited to vacuum atmosphere and, usually, radiation doses lesser than 1 MGy. The CoF of MoS_2_ increases considerably due to oxidation (from adsorbed water vapor or an oxidizing environment^148,157^) because the resulting molybdenum oxide (MoO_3_) particles demonstrate abrasive behavior.^151,156,158^ Since irradiation may increase oxidative reactions,^159−162^ the expected lifetime of MoS_2_ is significantly worse in these situations than in conventional applications. The presence of atomic sulfur and the susceptibility of MoS_2_ to radiation damage are additional limiting factors. Under irradiation, sulfur atoms are preferentially sputtered from the atomic lattice,^163^ which may cause stress-crack corrosion on steel substrates.^164^ Potential damaging effects and susceptibility to oxidation render unmodified MoS_2_ and similar transition-metal sulfide lubricants (e.g., tungsten disulfide) unfit for applications in environments with annual absorbed doses exceeding 100 kGy.^119^ An example of overcoming this seemingly fundamental challenge is the application of nanocomposite structures of MoS_2_/yttria-stabilized zirconia (YSZ) and thermal-annealing processing. The resulting nanograin stabilization leads to the inhibition of cracks and void accumulation while promoting self-adaptive lubrication^165^ under ion-irradiation conditions, suggesting the potential applicability of composite nanomaterials in the nuclear-reactor industry.

#### Graphite

Unlike MoS_2_, solid graphite films perform well in moisture-rich atmospheres, which aid in lamellar shearing and maintain lubricating properties.^148,151,156^ Efficient in temperatures of up to 400 °C,^151,166^ graphite is considered to be a generally stable and inexpensive dry lubricant,^151^ although it is not recommended for dry and vacuum environments. The effects of radiation on graphite are expressed through an increase in porosity, surface oxidation, crystallinity changes, amorphization, and deformation or minor changes in geometrical dimensions.^83,167−169^ In composite graphite materials and bulk volumes, radiation damage may lead to defect propagation and cracking, which induce a deterioration of mechanical properties.^167^ On the atomic scale, graphite starts to accumulate lattice defects at relatively low-dose irradiation (annual absorbed doses in the range 1 Gy to 100 kGy).^124^ Higher absorbed doses, reaching MGy levels, lead to a progressive transition from graphitic sp^2^ to diamond-like sp^3^ bonds,^83,168^ a gradual reduction of thermal and electrical conductivity, and potential swelling and warping of the material.^168^ Interestingly, the self-healing and structure recovery, typically achieved through thermal annealing at high temperatures (800–1300 K^168^), start to spontaneously emerge with the introduction of moderate levels of radiation doses (up to 200 kGy).^89^ This effect is caused by atomic collisions impacting local temperature increases, leading to crystalline structure recovery.^89^

Experimental data on low-dose-irradiated graphite suggests that the annealing processes within the graphitic crystal lattice are effective at low radiation dose rates (1.8 kGy/h^89^) and less impactful at higher dose rates.^123^ This finding suggests that graphite’s usability in high-radiation environments is determined by both the dose rate and the total absorbed dose. This means that graphite (and relevant carbon materials such as graphite flakes,^123^ graphene,^128,170^ and carbon nanotubes^89^) is potentially functional and usable in MGy dose ranges due to the partial restoration of accumulated damage.

Recently, Bradley et al. found an interplay between radiation-induced defect generation and defect annealing.^90,127^ However, the effects of high-radiation damage on solid graphite films and their tribological properties have not yet been experimentally studied. Based on the existing knowledge of graphite crystalline transitions in radiation fields,^127,168^ it is expected that graphite will retain most of its lubricating properties in both low- (<200 Gy) and potentially high-radiation (1–5 MGy) applications,^123,127^ depending on the specific work conditions, working temperature, and the binder chemistry of the dry-film lubricant.^153,171,172^

#### Hexagonal Boron Nitride

Boron nitride (BN), and especially its hexagonal form (hexagonal boron nitride - hBN) is yet another widely used dry lubricant material. Composed of lamellar, hexagonal crystals of alternating boron and nitrogen atoms, it is characterized by excellent electrical insulating properties, high oxidation resistance, and chemical inertness at elevated temperatures.^146^ This means that it is usable in high-temperature environments, and it is often applied on ceramic surfaces.^173,174^ However, due to its low wettability and relatively low adhesiveness, its wide application as a dry-film lubricant is limited.^151^ Under radiation, hBN undergoes damage akin to that seen in graphite. Sheets of hBN have been observed to deteriorate after exposure to a total neutron fluence of 3.5 × 10^16^ cm^–2^ using a flux of 2.40 × 10^12^ cm^–2^s^–1^, leading to a partial transition from sp^2^ to sp^3^ hybridization, local formation of cubic BN crystals, and amorphization. Similar to graphite, radiation-induced changes in crystalline structures degrade the electrical (dielectric) properties of hBN.^175,176^ In radiation applications, BN materials are considered to be some of the most effective choices for shielding and neutron capture^177^ due to the high thermal-neutron absorption cross-section of boron and the hydrogen-bonding affinity of hBN.^178^ This could make them more radiation sensitive in an environment in which high thermal neutron fluxes are present. The tribological performance of hBN in radiation applications has not yet been investigated in academic research; however, given its already broad application in aerospace, medical, and other industries,^177^ lubricating hBN films are promising candidates for further study and potential use in high-radiation fields.

### 4.2 Nanomaterial-Based Dry Lubricants

Compared to their bulk forms, nanostructured materials show significantly improved friction and mechanical properties; the nanometric dimensions allow these materials to be successfully used in both macro- and nanoscale systems.^147^ Nanostructured coatings, due to their fine size, inherently exhibit better adhesion and conformality than conventional materials when deposited on a substrate,^147,179^ even in the presence of pronounced surface roughness. Lamellar-structured materials—such as graphene derivatives, flake or few-layer MoS_2_, and hBN—are especially efficient thanks to the van der Waals interactions at the nanoscale and their flexibility, making thin, lubricating films of these materials applicable for both soft and hard surfaces.^180^ Application of nanomaterials is especially crucial in boundary lubrication regime, i.e., under operating conditions where oils and greases cannot form a stable lubrication film to fully separate the surfaces (e.g., in space applications or MEMS devices^181−184^). Since grain boundaries can act as defect sinks^106−109,185,186^—trapping mobile, diffusing defect cascades created under heavy irradiation^106^—materials exhibiting nanoscale grain sizes are especially durable in radiation applications. However, the core chemical and crystalline properties of materials observed in their bulk form are still present at the nanoscale, thus making some interesting nanomaterials potentially problematic for high-radiation applications (e.g., MoS_2_ being susceptible to oxidation^119^ or radiation-damage prone fluorine based materials^112^).

#### Carbon-Based Nanomaterials

Carbon-based nanomaterial lubricants have been extensively studied in recent years.^2,187^ As a general example, nanostructured coatings based on graphite nanoparticles have been found to directly improve load-bearing capacity, wear protection, and increase contact stiffness and elastic capacity.^187^ An extremely low CoF—i.e., of the order of 10^–3^,^187,188^ also known as superlubricity—is achievable by depositing graphite nanoparticles; this improves the surface coverage of the coating on areas of micro- and nanoscale roughness and smooths the working surfaces at the atomic level. Even though nanoscale graphite offers better lubrication, it is expected to have similar radiation stability to its bulk, macroscopic form.^123^

Graphene—single-atom-thick sheets of sp^2^ carbon atoms—shows high chemical inertness, low surface energy, easy shear capability, and an atomically smooth surface, which together result in tribological properties that surpass those of graphite. In contrast to bulk graphite, the performance of graphene is independent of the working environment; it does not rely on a humid atmosphere to maintain lamellar shearing and lubrication.^187,189,190^ Moreover, it has been demonstrated that graphene-based coatings, due to their impermeability, protect surfaces from corrosion, slowing down oxidative processes.^189,191^ The rise of graphene fabrication and synthesis methods in recent years has paved the way for applying graphene coatings on both micro- and macroscale devices, making it an extremely versatile material for most working environments.^2,192−196^

Under radiation, as with graphite, the graphene lattice is susceptible to knock-on collisions and carbon-atom displacement.^197^ Displaced atoms can recombine back into the sp^2^ or sp^3^ lattice, forming defects in the crystal structure. Heavy-ion irradiation studies have demonstrated defect-healing effects occurring in a low-fluence and low-energy regime.^198^ At high energies (irradiation of 10^14^ ions/cm^2^ of 150-MeV Au ions^199^), graphene tends to partially amorphize due to radiation damage, although how this transition influences tribological properties has not yet been experimentally investigated. Similar effects—i.e., an initial decrease followed by the restoration of crystallinity—have also been observed with MGy-range gamma irradiation.^128,129^ Interestingly, in contrast to bulk graphite, graphene planes are able to dissipate the energy from radiation-induced collision cascades (e.g., on the substrate/coating interface), reducing the damage to the system,^200^ which means that it is potentially suitable for high-radiation applications.

Diamond and diamond-like films are hard materials composed of either nanocrystalline diamond (NCD; a nanostructured form of diamond with only sp^3^ carbon atoms) or diamond-like carbon (DLC; an amorphous structure comprising a mixture of sp^2^- and sp^3^-bonded atoms sharing some core properties with diamond crystals).^201−203^ DLC is currently being studied as potentially interesting for friction-reduction and wear-protection applications; it is also a promising candidate for use in radiation environments. However, even though bulk diamond crystal is considered relatively radiation resistant,^204^ under heavy-ion irradiation, DLC films have been shown to perform poorly due to radiation-induced graphitization,^205,206^ which leads to a gradual decrease in lubricating properties. Interestingly, due to their dense grain-boundary structure,^205^ NCD films do not lose their tribological properties despite similar radiation damage. It has been shown that under heavy-ion irradiation, the structural conversion of NCD leads to the formation of a quasi-stable composite-layered structure, which enhances lubricating properties. In a gamma field, NCD films have been tested up to 300 Gy;^207^ this makes them a viable candidate for low-radiation applications and provides motivation for their further study.

#### Composites and Nanocomposites

Using composite materials reinforced with lubricious nanoparticles could help achieve long-term lubrication in extreme working conditions where frequent maintenance is impossible. The effects of adding lubricating nanoparticles to metal, ceramic, and plastic materials have been widely studied,^181,208−210^ and it can lead to significant improvements in CoF and wear resistance. Interestingly, some nanomaterials are already widely studied to promote radiation resistance in structures such as ceramic YSZ.^165,211^ Embedding lamellar particles (MoS_2_, graphite, graphene, hBN) within a material can enable self-lubrication through continuous release of lubricious nanoscale flakes, dynamically filling asperities along the working surface.^208^ Zero- and one-dimensional nanoparticles (e.g., fullerenes, nanotubes, and nanorods) can improve tribological properties through a self-polishing effect and by acting as nanoscale roller or ball bearings. The addition of both BN nanotubes^212^ and carbon nanotubes^213^ to metal alloys was found to enhance their hardness and yield strength, leading to improved wear resistance and radiation-shielding performance.

Graphene powder has been added to plastics, polymers, and metals to promote self-lubrication and aid energy dissipation.^200,209,214^ Interestingly, similar to some wet lubricant systems, specific material compositions may have synergistic effects in low- and moderate-radiation systems; for example, a PTFE/graphite composite has been found to exhibit an improvement in CoF after 150 kGy gamma irradiation due to graphite fluorination.^142^

Nanoparticle-infused solid composites enable a fine-tuning of properties and represent an attractive opportunity for developing tailored, long-lasting, self-lubricating, and wear-resistant materials for a broad spectrum of radiation applications in which human access is limited. Nonetheless, radiation tolerance remains highly dependent on the bulk matrix material. One has to be aware of the risk of releasing the nanoparticles from their matrix during operation, potentially creating highly volatile and possibly radioactive or hazardous dust. Any potential dry lubricant materials should therefore be carefully analyzed in the context of their working environment and absorbed radiation doses. Because of the limited availability of radiation-aging data, the lifetimes of lubricants, the changes in their tribological properties, their mechanical and chemical stability, and their working safety are challenging to evaluate.

## 5 Wet Lubricants

Due to their versatility, wet lubricants are the most common type of lubricating material used in modern machinery.^215,216^ In a radiation environment, however, their use is limited by their radiation tolerance, which poses an open challenge to engineers and researchers.

### 5.1 Base Fluids

Typical lubricating oil is composed of long hydrocarbon chains, with additional potentially nonhydrocarbon elements and other functional groups attached to the main chain. The effects of radiation on these systems depend on the specific chemical family of each of the oil’s components, including the base fluid and any additives. Furthermore, in some cases, significant differences between materials and products belonging to the same chemical category have been reported.

**Figure 6:**
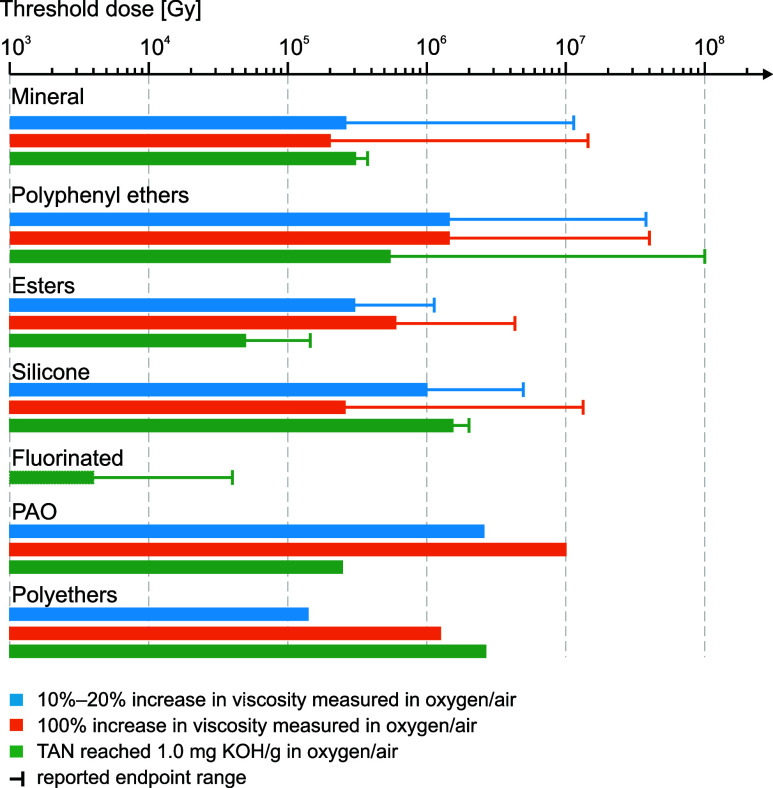
Radiation tolerance of oil products based on their base-fluid composition, compiled from estimated radiation dose thresholds according to the measured increase in viscosity and observed TAN in air or oxygen. Data were estimated using available research data on mineral,^15,43,60,78^ polyphenyl ethers (PPEs),^13,25,42,60,71,74−78^ ester,^60,217^ silicone,^13,60,77,78^ fluorinated,^60^ PAO^60^ and polyether-based oils.^60^

Figure 6 presents a summary of the results of experimental studies reporting data collected on irradiated lubricants from the 1960s to the present day. Typically, two different properties are used to characterize oils as a function of the total absorbed dose: viscosity and TAN; these are considered to compare the macroscopic effects of radiation damage. The bars shown in the graph represent dose values associated with specific radiation-induced modifications of viscosity and TAN, referred to as end points. The selected end point for viscosity corresponds to a variation of 10%–20% and 100% with respect to the nominal unirradiated value; the TAN end point corresponds to a value of 1.0 mg KOH/g. Variations and differences between the reported end point values shown in Figure 6 stem from comparing different data sets and test results performed under varying conditions.

Based on the available data, oils are categorized by their chemical family: mineral oils,^15,43,60,78^ polyphenyl ethers (PPEs),^13,25,42,60,71,74−78^ esters,^60,217^ silicones,^13,60,77,78^ fluorinated oils,^60^ polyalphaolefins (PAOs),^60^ and polyethers.^60^ Apart from fluorinated oils, which are considered the most radiation sensitive and reach end points between 2 and 50 kGy, the other oils show a wide range of radiation tolerance, overall ranging between about 30 kGy and a maximum of 30 MGy. Based on experimental evidence, PPE-based oils are the most radiation resistant. Large variations of about 2 orders of magnitude are reported in Figure 6 between oils belonging to the same family, for example, among the mineral oils.

Interestingly, research has also shown large variations between the end points associated with viscosity and TAN. Most oils, such as PPE- and mineral-based oils, are, on average, more sensitive to TAN variation than viscosity, but the opposite is true for polyether oils. This variance strongly depends on the detailed chemical composition differences between products and the irradiation conditions of the performed studies. It is, therefore, important to understand a broad context of typical irradiation tests and the nature of complex radiation-damage phenomena to analyze and predict lubricant lifetimes in high-radiation areas.

It is noted that the studies presented here were performed under differing irradiation conditions; for this reason, systematic comparisons taking into account irradiation parameters other than the total absorbed dose are not possible. For the compilation of Figure 6, the used dose values are the ones reported in the cited literature, as further discussed in Section 6.7.

The subsections that follow present further discussion of the various oil categories.

#### Phosphates and Halocarbons

Since the resistance of each molecular compound can be roughly approximated by the resistance of its weakest molecular bond, it is known that the oil families least resistant to radiation are the phosphates and halocarbons. These include fluorinated products, which tend to evolve corrosive acidic products,^3,218^ as demonstrated by high TAN values.^3,60,104,219^ It has been reported that about 20% of phosphate esters are converted to acids after absorbing 1 MGy in γ radiation,^3,104^ resulting in a TAN of around 10 mg KOH/g. High TAN values were also reported in the case of fluorinated oils, for which values between ∼2 and ∼30 mg KOH/g were obtained after gamma irradiation up to ∼0.9 MGy at 10 kGy/h in contact with air.^60^ Additionally, high acidity levels were found to yield extensive corrosion of metal surfaces in contact with the irradiated samples.^3,104^ Based on the maximum recommended TAN values (0.3–3.0 mg KOH/g^220,221^), the resistance of some perfluoropolyether products, for example, would be about 50 kGy in a gamma environment at room temperature and in vacuum.^60^ This means that phosphate and halocarbon oils are generally not compatible with radiation applications and may indeed be hazardous in such situations.

#### Silicone-Based Oils

Despite keeping their oxidation stability after absorbing 1 MGy,^3,104^ aliphatic silicone-based fluids are reported to be sensitive to radiation-induced viscosity increases.^222^ The gelation point, which is defined as the dose at which a cross-linked fluid becomes essentially a solid, is highly dependent upon the molecular weight of the silicone fluid; it has been reported to range between 0.05 and 15 MGy for different oils.^3,223^

Increased radiation tolerance has been reported for aromatic silicone oils, which show limited viscosity variations up to 10 MGy of dose under different irradiation conditions.^77^ As in the other radiation-resistant oil examples, the aromatic component is likely responsible for this improved tolerance.

#### Ester-Based Oils

Ester-based lubricating oils are reported to have limited radiation resistance, especially if they contain phosphate or silicate functional groups. Irradiation of dialkyl sebacates up to 1 MGy in γ radiation in air at room temperature typically yields a viscosity increase of about 35%, a TAN increase of ∼7 mg KOH/g and a reduction in flash point of almost 90 °C.^3,224,225^ The only exception is aromatic esters such as dialkyl terephthalate, for which a similar drop in flash point was observed only after 9 MGy under the same irradiation conditions.^3,226^ Successful operational tests on a diester oils irradiated up to 0.3 MGy and used for the lubrication of a vacuum turbopump were reported by Beynel et al.^49^

#### Mineral-Based Oils

Mineral and synthetic oils have variable resistance to radiation, spanning from the kGy to the MGy range.^15,43^ In a few cases, they can reach tens of MGy,^78^ depending on factors such as their saturation, branching, molecular weight, and their paraffinic, naphthenic, and aromatic content. At equivalent branching and molecular weight, saturated molecules tend to evolve more hydrogen than unsaturated ones but are more resistant to cross-linking.^103^ Linear molecules predominantly release hydrogen, while the production of light-hydrocarbon gases increases with molecular branching,^103^ potentially leading to the creation of damaging acids or excessive bubbling. Higher molecular weight is usually synonymous with a lower gelation point.^3,227^ Paraffinic compounds are generally reported as being less resistant than naphthenic ones, which are in turn less resistant than aromatics.^60,103^ This classification is based on the rate of viscosity changes, amount of evolved gas, and rate of oxidation.^15,43^

#### Polyphenyl Ether-Based Oils

As noted, the radiation tolerance of organic polymers is overall attributed to the superior resistance of the aromatic ring—a main component of PPE molecules. PPEs are widely regarded as the most resistant organic structures used in formulating lubricants.^71,72,219,228^ It has been observed that pure PPEs produce significantly fewer gas products than aliphatic hydrocarbons (a reduction of up to 100 times^60^). To improve lubrication performance, PPEs must often be combined with alkyl chains at the expense of their radiation tolerance. In general, the lower the aromatic content over alkyl groups in terms of the number of C atoms, the greater the radiation-induced changes in viscosity, TAN, and radical yield in both vacuum and bubbling-oxygen conditions. Dose thresholds are two to five times higher in oils with similar composition but greater aromatic content.^77^ The exact formulation critically impacts radiation tolerance, as large variations in the effects of radiation are associated with different mixtures of the same set of PPE-based oils, as shown in Figure 6.^72,75^

PPEs are reported to remain liquid after absorbing doses ranging from a few MGy to a maximum of 100 MGy.^219^ Viscosity changes of less than 100% are reported for many formulations at doses ranging between 1 and 10 MGy; much higher viscosity increases of 800% to 2000% are reported for some formulations at doses exceeding 20–30 MGy. PPEs are reported to be generally resistant to oxidation and show TAN values limited to a few mg KOH/g at 10 MGy;^60,75^ their pour points remain below 0 °C after irradiation.^77^ Regarding the operation of lubricated equipment, failures of motors lubricated with PPE oils attributed to lubricant solidification or general lubricant degradation are reported to occur between 30 and 70 MGy.^13,76^

To date, there is no evidence of any other formulations exhibiting comparable overall performance to PPE oils under radiation at doses exceeding the 10 MGy range (Figure 6). In light of this, they are the most commonly used materials for formulating radiation-resistant greases, as discussed in Section 5.3. Overall, PPE-based products constitute the primary category of materials that have been systematically irradiated, tested, patented, and selected for use in high-radiation areas over the past few decades.

### 5.2 Additives

Most commercial wet lubricants contain additives to improve the performance of the base fluid; however, as these additives are typically organic, they may also be sensitive to radiation themselves. Depending on their resistance relative to the base oil and the overall resistance of the produced mixture, additives can either retard radiation damage in a lubricant, become ineffective and be used up during irradiation, or accelerate damage.^3^ The impact of additives on the functional properties of lubricants after irradiation is not completely understood and is still being debated.^43^ Considerations of the main categories of additives whose radiation resistance has been investigated are reported in the subsections that follow. Additives that are typically used for the formulation of greases, such as thickeners, are discussed in Section 5.3

#### Antioxidants

Interaction with oxygen usually has a negative effect on lubricant lifetime because oxidation promotes an increase in viscosity and creation of harmful products such as acids, affecting performance and potentially damaging the lubricated components. Antioxidants, which have the role of neutralizing radicals and stopping oxidation chain reactions, are therefore some of the most widely used additives. Unfortunately, common antioxidants such as amine compounds or tricresyl phosphate (TCP) have been shown to be very sensitive to radiation.^3,104,224,225^ Interestingly, selenide-, sulfur-, and iodine-containing antioxidants (e.g., dialkyl selenide, disulfide, and phenothiazine) have been shown to be effective up to ∼1 MGy, especially when present in aromatic forms.^3,103−105,224^ It is important to note that oxidation inhibitors are more effective in less-resistant oils (e.g., esters and aliphatic hydrocarbons) than in more radiation-tolerant ones such as PPEs,^3,104^ and that a noninhibited PPE irradiated up to 28 MGy with electrons is still more resistant to oxidation than an inhibited fresh diester.^104,219,228^ By creating free radicals that readily react with oxygen, radiation accelerates oxidation, leading to a rapid depletion of antioxidants. Antioxidant concentrations of the order of 10% have been shown to be necessary to inhibit the effects oxidation accumulated up to 1 MGy.^3,104^ In the simultaneous presence of oxygen and radiation above 1 MGy, it is therefore recommended to use base oils with superior intrinsic oxidation resistance.^219,228^

#### Viscosity-Index Improvers

Viscosity index (VI) improvers are commonly used in lubricating oils operating over a wider temperature range. These long polymers stretch out at higher temperatures to partially compensate for oil thinning. Bolt and Carroll described the opposing effects of radiation on different types of VI improvers^3,105,226,229^ and patented tailored combinations of oils and additives for optimizing the stability of viscosity under irradiation.^230^

#### Anti-Foam Additives

The use of antifoam additives in lubricants seeks to limit the formation of foam from trapped air, which would result in increased wear. Typically, an addition of around 0.01% of a silicone-based fluid is used for this purpose.^3,220^ Such additives are very sensitive to radiation,^3,60,219,224,225,231^ and they have been reported to become ineffective as defoamers after ∼0.1 MGy.^225,226^ No radiation-resistant alternatives have yet been identified, which is concerning given that radiation increases foaming via gas evolution.^3,226,231,232^

#### Antiwear Additives

Lubricants for highly loaded mechanisms operating in the boundary regime employ extreme pressure (EP) and antiwear (AW) additives to limit surface damage. These additives create a mechanically resistant layer by chemically reacting with the metallic surface. Sulfur- and chloride-containing compounds are common constituents of EP additives, while phosphates have AW properties. These additives have generally been found to be less radiation resistant than base oils.^224^ This is especially true for chlorine-based additives and TCP, which have been reported to produce corrosive acids under irradiation.^104,218,233^ The effects of radiation on zinc dialkyl dithiophosphate, a very common AW additive,^234^ have not yet been studied or published. Similarly, there have been no reports on the impact of radiation on dispersants and other additives that prevent the accumulation of pollutants and sludge by encapsulating them through their polar and nonpolar ends.

#### Solid Lubricants

In cases of intermittent motion, extremely high loads, or fluctuations in oil viscosity, optimal elastohydrodynamic lubrication may not be achieved. During operation, surfaces are typically in direct contact (the boundary lubrication regime^235,236^). In addition to the use of stress-activated additives,^237^ tribological properties can be improved using solid lubricants that fill the volume between surface asperities and effectively reduce surface roughness. Therefore, adding specific solid particles to oils can substantially improve their lubricating properties.^145^ Typical solid lubricants include graphite, MoS_2_, and PTFE, the radiation resistance of which has been discussed in Section 4. It is noted that if solid additives are present in oil-based lubricants, solid–liquid interfaces form within the bulk volume in addition to the mechanical surfaces, potentially intensifying the effects described in Section 2.2.

### 5.3 Greases

Greases are semisolid materials containing, on average, 85% base oil fluid, 10% thickener, and 5% other additives.^216^ Various thickening agents can be added to liquid lubricants to produce semisolid final products. Common thickener types include metallic soaps, organic compounds such as polyurea or PTFE, and inorganic materials.

As greases are multiphase mixtures, their response to radiation arises from the complex chemical–physical interactions between their components. Given the relative ratio of oil to thickener content, the strength of their interaction is somewhat reflected in the resulting grease consistency. Consistency is typically measured through the depth of penetration of a free-falling cone into the bulk surface of the grease when housed in a standard container geometry and for a determined duration (known as the cone penetration test). Consistency is one of the standard and most commonly reported properties of grease in irradiation tests. Before such a test, grease is usually worked using a specific handler that has holes through which it is forced to repeatedly pass. Worked-consistency variations within 10%–15% are generally accepted as stability end points for greases.^3,42,71,73,74,103^

**Figure 7:**
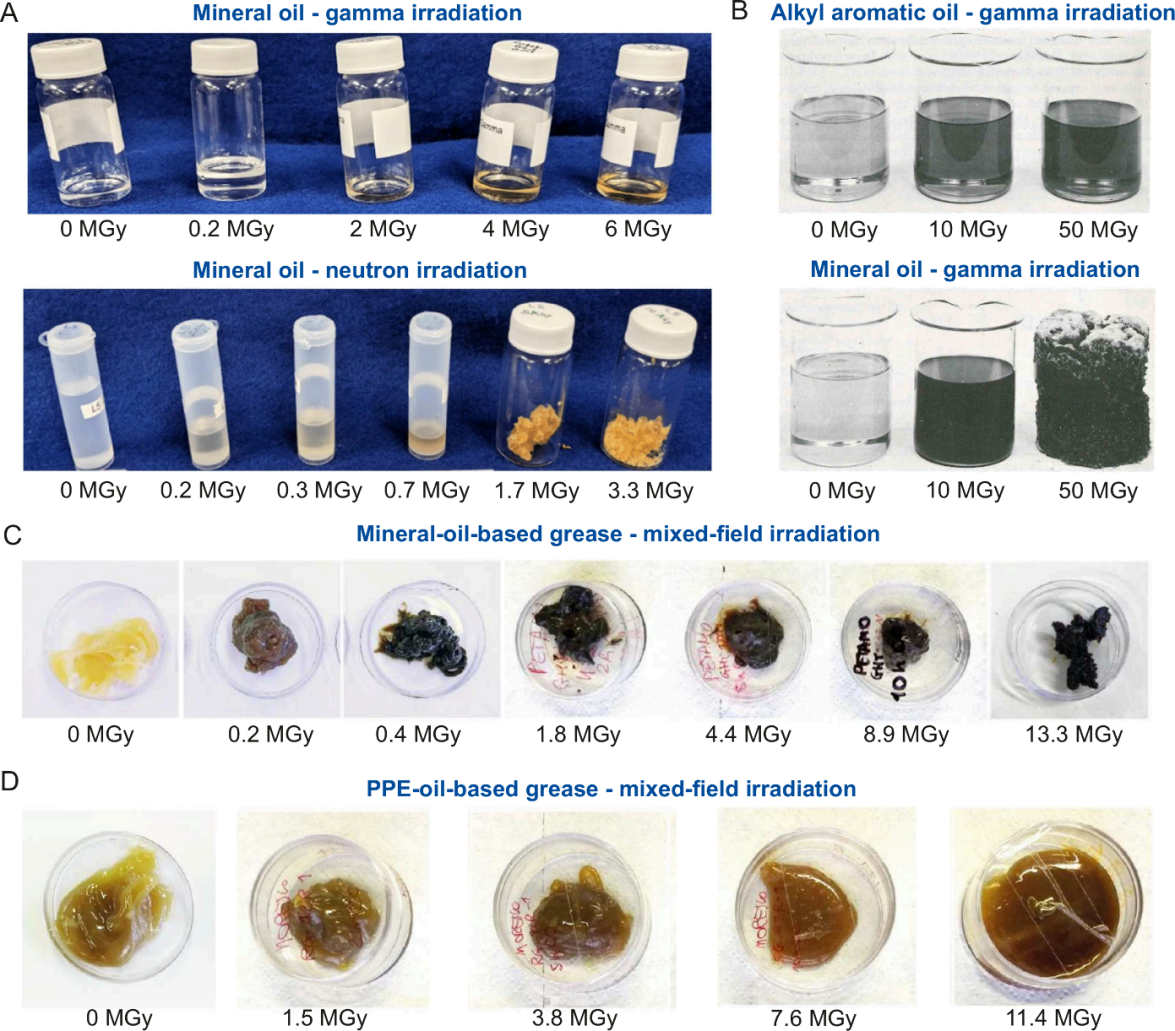
Examples of damage caused to oils and greases by high-dose radiation. (A) Macroscopic effects of radiation on mineral oil: oxidation-induced discoloration and gradual cross-linking occur in a gamma field, while brittleness develops with increasing neutron irradiation dose.^15^ (B) Depending on the chemistry of the base oil, the effects can vary drastically, from changes in color or rheological properties to complete loss of the initial liquid state.^3,229^ The effects of radiation damage are more complex in greases, in which degradation of the properties of the base oil and the thickeners may occur at different rates, leading to different macroscopic results of (C) hardening or (D) softening.^25^ Panel (A) is adapted from Bliznyuk et al.^15^ Available under a CC-BY license. Copyright 2023, MDPI. Panel (B) is adapted with permission from Bolt and Carroll.^229^ Copyright 1959, Taylor & Francis. Panel C and D are adapted from Ferrari et al.^25^ Available under a CC-BY license. Copyright 2021, Elsevier.

So far, two main and competing macroscopic behaviors have been observed in greases, depending on the relative radiation sensitivity of the base oil, the thickener, and the gel structure originating from their interaction.^74^ If the radiation-induced damage to the thickener structure or surface properties prevails, the grease progressively softens, and oil separation can occur; in the worst case, this can lead to liquefaction.^3,42,74,103^ Grease softening is one of the most often reported macroscopic effects of radiation.^3,42,79^ If, by contrast, the prevailing effect of radiation is oil cross-linking, the grease will harden and eventually solidify; in the most extreme cases, it cannot be tested after irradiation due to a dramatic change in properties.^25,42,74^ Such effects are visible to naked-eye investigations and are evident when irradiated greases are handled, as shown in Figure 7.

A radiation-resistant grease should, therefore, ideally contain an oil and thickener that are both radiation resistant. Some authors suggest that stable greases can be obtained by combining ingredients that exhibit mutually compensating consistency changes under radiation.^103^ Supposing that the radiation tolerance of greases can be mainly based on cone penetration, Arakawa et al. showed that suitable thickeners could be used to realize greases with much higher radiation tolerance than the base oil.^74^ However, the use of a single property to characterize the radiation stability of materials has limitations, as will be further discussed in Section 6.3. As shown in Figure 6 for fluid oils, different dose thresholds are identified when using different end points, such as viscosity and TAN.

Historically, greases have been systematically tested less frequently under radiation than oils, as it was believed that their radiation tolerance depends predominantly—or even uniquely—on the radiation tolerance of the oil used for their formulation.^3,78^ However, comparative studies on greases manufactured with the same base oil and different thickeners have shown important differences in the assessed stability, up to 1 order of magnitude in dose.^71^ Radiation-induced changes to the worked consistency also seem to be critically linked to the type of thickener.

**Figure 8:**
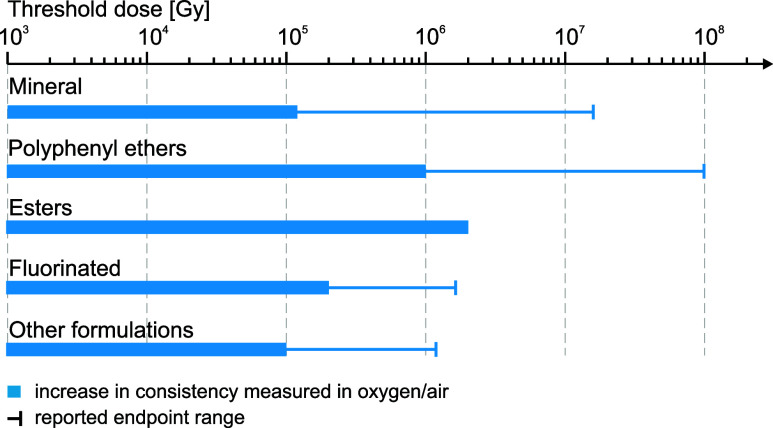
Compilation of estimated radiation dose thresholds for greases based on consistency changes. Data were estimated using available research on mineral-oil-,^3,25,42,71,73,74,79^ polyphenyl-ether-,^9,11,12,14,72−74,78^ ester-,^78^ and fluorinated-oil-based greases^25,42^ along with other unspecified formulations.^25,42^

Figure 8 summarizes the results of the available studies reporting consistency and texture analysis of irradiated greases. Worked consistency was analyzed to characterize these greases as a function of total absorbed dose. The bars in the graph represent dose values associated with 10%–15% consistency variation end points or dose values associated with a complete change of grease texture, corresponding to fluidization or hardening, invalidating the consistency test. Based on the available data, four main categories of grease are reported depending on the composition of their base fluids: mineral oils, PPEs, esters, and fluorinated oils. For completeness, other unspecified formulations are also reported in a single grouped category.

As already observed for oils, fluorinated greases are the most radiation-sensitive products, reaching end points between about 0.2 and 2.0 MGy. Mineral-oil-based greases exhibit a wide range of dose thresholds, from 0.1 MGy and exceeding 10 MGy in a few cases. In alignment with the expected radiation resistance of base fluids, the most radiation-resistant category of greases is represented by PPE-based formulations, all exceeding 1 MGy of tolerance and reaching an outstanding value of 100 MGy in some reported cases.^74^

Comparing Figures 6 and 8, on average, it seems that oils are more radiation sensitive than greases having similar formulations. However, the quantities used to determine end points for oils and greases are not the same, so the dose thresholds are not directly comparable.

Significant variations of more than 2 orders of magnitude have been reported in the resistance of both mineral and PPE-based products. These discrepancies within the same chemical family can be attributed to a combination of the intrinsic and extrinsic aspects of the irradiated samples and their characterization. Intrinsic parameters include the exact composition, structure, nature and amount of additives, production process. A large variability in performance results even before considering radiation. It should be noted that these intrinsic details are typically confidential, making any estimates of performance under radiation challenging. Extrinsic parameters concern the irradiation conditions that depend on the design of the given irradiation test, on the chosen setup and on the specific combination of radiation, temperature, atmosphere, and other relevant parameters. Last but not least, the postirradiation characterization methods, definition of usability thresholds and classification in chemical families can introduce apparent discrepancies requiring additional details for their better understanding. Here above, greases have been grouped based on the chemistry of their base oil, for the sake of consistency with Section 5.1. Most studies on irradiated greases assess their usability threshold based on worked cone penetration, the value of which can be significantly affected by radiation damage to the thickener. Greases containing the same type of base oil but different thickeners could, therefore, be attributed to dissimilar levels of radiation resistance despite being classified here within the same family. These results raise awareness of how products having apparently similar compositions can have incredibly different and unpredictable lifetimes in operation.

#### Metallic Soaps

Metallic soaps are one of the most common types of thickener used in lubricating greases, and lithium, calcium, and aluminum soaps are the most common thickening agents currently on the market.^215^ The long hydrocarbon chains in metallic soaps are sensitive to ionizing radiation. As radiation progressively breaks these chains,^3,61^ this leads to oil separation, grease softening, and liquefaction. Conventional greases based on aliphatic metallic-soap thickeners have been reported to undergo critical structural changes between 0.1 and 1.0 MGy, as evidenced by changes in cone penetration measurements^3,42,103^ or rheology.^71^ Aluminum soaps have been found to be marginally more resistant than sodium, lithium, and calcium soaps; however, they are less likely to recover their consistency through annealing.^3,61^ In the presence of carboxylic acids produced by the radiolytic oxidation of hydrocarbon base oils, the degradation of soap thickeners is further evidenced by the release of oxygen-containing gases.^71^ The presence of aromatic rings within metallic-soap thickeners is linked to greater stability in worked penetration, drop point, and gel strength,^218,233^ especially when combined with aromatic oils.^231^

#### Inorganic Thickeners

Greases formulated with inorganic thickeners, such as silica and bentonite, have been reported to exhibit strong resistance to radiation-induced softening.^3,79,103^ Methane production has been reported in silica-based greases after exposure to 0.36–3.6 MGy of γ radiation, likely due to the degradation of silica surface modifiers;^71^ however, no significant effects on the structure and rheology of the grease have been observed. Given their promising radiation tolerance, these inorganic thickeners have been combined with PPE oils for the manufacture of specialty greases that can remain stable from 3.6 MGy up to 100 MGy in different irradiation conditions.^42,71,74^ It has been reported that consistency changes remain within 10%–15% even after 30–100 MGy of gamma irradiation^73,74^ and ∼12 MGy in a mixed field.^25,42^ Petroleum-based formulations used for comparison, such as lithium or silica gel, were reported to liquefy at 5 MGy of gamma dose when irradiation was performed at high temperature and to both solidify at 50 MGy of gamma dose when irradiation was performed at room temperature.^74^ Greases based on PPE oils with inorganic thickeners have also shown stability under the combined effects of radiation and temperature.^71^ PPE-based greases used for the lubrication of ball bearings and motors studied for the construction of the ITER were found to be resistant, based on equipment functionality, up to about 16 MGy.^10^

The addition of aluminum flakes to a PPE-based grease formulation was reported by Onishi et al.^11^ to improve its radiation tolerance from 60 MGy to the extremely high dose level of 100 MGy. In their study, the operation of a motor lubricated with this grease was used as an indicator of its radiation tolerance. In addition, based on their established radiation tolerance, greases formulated with PPE oils and inorganic thickeners have recently been used for the lubrication of critical equipment in the CERN accelerator complex,^24,25^ and they are currently under further investigation.^52,54^

Combining inorganic thickeners with mineral oils also leads to greases with superior stability as measured via cone penetration test. A patent from 1966^79^ reports the stability—based on worked and unworked consistency—of a grease composed from equal weights of mineral lubricating oil and lead silicate material up to a gamma dose of 1 MGy. This grease was reported to be more stable than other mixed formulations. Tests performed at CERN and the Atomic Energy Research Establishment (Harwell, UK) on a selection of greases based on mineral oils and inorganic thickeners showed stability up to a dose of 10 MGy.^78^

Greases based on hydrocarbon oil and bentonite or silica resisted 5 and 10 times longer, respectively, than their complex sodium-thickened equivalent. The use of an inert atmosphere (argon) during irradiation was shown to considerably retard damage, as further discussed in Section 5.4, bringing the resistance of the bentonite grease to 1.3 MGy under the studied irradiation conditions.^71^

#### Other Thickener Types

Despite its polymeric nature, polyurea has been reported to be resistant to high levels of radiation due to its stable urea bonds.^3,25,42^ A 15% change in worked penetration was found to occur after about 4 MGy in gamma;^103^ similar stability in worked penetration was reported up to 8.9 MGy absorbed in a reactor mixed field.^42^ Cone-penetration tests of a silicone grease thickened with aryl urea found stability within ±60% of the original value after exposure to 9 MGy of γ radiation, similarly to terephthalamate, while lithium stearate and carbon black reached ±100% in the same test.^223^

Highly aromatic dyes constitute another type of thickener with exceptional radiation resistance. For example, copper phthalocyanine was found to perform the best among the compounds studied by Fischer et al., with ±60% penetration stability preserved up to 14 MGy.^223^ Indanthrene has also gained particular interest after resisting the combined effects of 8 MGy electron irradiation, oxidation, and mechanical working.^105^

Plastics such as polypropylene or PTFE can also be used as grease thickeners.^215^ Degradation of their mechanical properties under radiation has been reported;^49,238^ however, the consequences on the performance of the grease have not yet been examined.

### 5.4 Effect of Irradiation Conditions

The specific irradiation conditions are known to play a role in determining the effects of radiation on organic materials. However, due to the technical difficulties of performing parametric studies, there are only limited data available that allow for consideration of the roles of specific parameters.

#### Effect of Oxygen

Only a few studies have systematically investigated the differences between irradiating under inert (argon, vacuum) and oxygenated (air, pure or bubbling oxygen) atmospheres.^60,71,77^ Additionally, the results are difficult to compare, as the materials, irradiation conditions, and quantities tested in these studies all differ. A rough estimate of the effect of oxygen on radiation damage can be obtained for a specific end point by defining the usability-threshold ratio as follows:
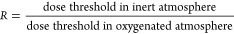
A few examples of *R* compiled from the available data are listed in Table 1.

**Table 1:** Usability-Thresholds Ratios *R* Obtained after Irradiation under Inert and Oxygenated Atmospheres

**Lubricant**	**Dose rate**	**Irradiation atmospheres**	**Studied property**	*R*
Mineral oil and bentonite grease^71^	0.36 kGy/h	Argon/air	Shear strength	10
Mineral oil and complex sodium grease^71^	0.36 kGy/h	Argon/air	Shear strength	3
Mineral oil and silica grease^71^	0.36 kGy/h	Argon/air	Shear strength	1.6
PPE oil 1^77^	10 kGy/h	Vacuum/O_2_	Viscosity	1.1
PPE oil 2^77^	10 kGy/h	Vacuum/O_2_	Viscosity	1.2
Mineral oil 1^77^	10 kGy/h	Vacuum/O_2_	Viscosity	1.05
Mineral oil 2^77^	10 kGy/h	Vacuum/O_2_	Viscosity	1.7
Silicone oil^77^	10 kGy/h	Vacuum/O_2_	Viscosity	0.66

When oxidation is not a significant degradation mechanism for the material but rather serves to inhibit other more damaging processes, irradiation in the presence of oxygen can lead to similar or lesser damage than in its absence,^66^ leading to *R* ≤ 1. However, accelerated degradation is generally reported when lubricants are irradiated in oxygenated atmospheres, this being especially true at low dose rates (for example <1 kGy/h),^71^ where *R* values can reach 10. At higher dose rates (>10 kGy/h), the effect of oxygen decreases;^60^ this is likely due to limited oxygen diffusion into the bulk material. This hypothesis is further supported by the very low levels of oxidation evidenced by Fourier transform infrared (FTIR) spectroscopy and TAN measurements of an oil irradiated at an extremely high dose rate of about 800 kGy/h,^75^ which led to hypoxic-like irradiation conditions. This demonstrates the importance of appropriate distribution of oxygen within the tested material to accurately represent oxidation-related damage in accelerated test conditions.

The effects of oxygen can be investigated by decreasing the dose rate, but this requires extremely time-consuming irradiations, especially to reach doses in the MGy range. Alternatively, it can be investigated by forcing oxygen diffusion through the material and selecting geometries and irradiation setups that allow for oxygen diffusion; thin sample layers are preferable for these investigations.^66^ The maximum effect is achieved at low dose rates/high permeability when the oxygen replenished is continuously in equilibrium with the active species of the irradiated polymer.^70^

Temperature and pressure also affect diffusion rates,^70^ and methods have been developed to optimize oxidation throughout a sample volume by applying pressurized oxygen atmospheres. Increasing the oxygen pressure at a high dose rate has been proven to result in an equivalent response to decreasing the dose rate at atmospheric pressure.^70^ Hydrocarbon-based greases exposed to radiation and temperature in the presence of atmospheric oxygen were found to undergo significant changes,^71^ possibly demonstrating the accelerating effect of temperature on oxidation and radiolysis.

Performing irradiation under oxidation conditions in bulk samples is technically challenging, since it is necessary to provide oxygen in a controlled way to the entire material volume while considering both the diffusion kinetics of oxygen in that volume and its consumption by radiation. The amount of oxygen initially present in a material seems to play a minor role in assessing the effects of oxygen, especially at high doses and high dose rates. By contrast, if the sample is thin enough and the dose rate is sufficiently low, new oxygen will diffuse into the material, replenishing that consumed during irradiation. Irradiation tests are typically conducted under accelerated conditions, so considering the effect of oxygen is crucial for extrapolating the results of such tests to the prediction of material degradation in actual operation.

#### Role of Radiation Type

In a meta-study of gas yield and viscosity in irradiated organic liquids, Collins and Calkins^227^ conjectured that radiation damage is independent of the dose rate and radiation type within a factor 2, provided that other parameters (such as temperature and atmosphere) do not play a role in the material degradation. However, while this factor may be considered negligible at low total absorbed doses, it becomes significant when materials are employed in conditions close to their usability thresholds.

Moreover, as detailed in the previous paragraphs, thermal and atmospheric conditions in general considerably impact the radiation effects on materials, and therefore Collins’ conjecture can rarely be employed. Unfortunately, many publications, among others^48^ adopted the ”equal dose = equal damage” conjecture without considering the conditions on temperature and atmosphere, and omitting the validity within a factor of 2. Other researchers^49^ emphasize the effect of dose rate, but neglect the effect of radiation field composition.

Subsequent experimental studies described in the following paragraphs prove that the ratio between radiation-induced damage in different radiation fields can exceed this value and increase up to 10 depending on the specific set of irradiation conditions, the measured quantity and the tested material. Despite the lack of strong experimental evidence, some researchers still apply this simplified claim to this day, both for pragmatic reasons and due to the difficulty of collecting data in radiation fields other than gamma.

Given the increasing requirements placed on materials in terms of radiation resistance, the use of different radiation types may produce apparent success in testing but lead to failure in actual operation. Therefore, it is important to understand the potential impact of different radiation-field conditions on damage to materials. Nonetheless, only a few studies have conducted comparisons of the effects of different radiation fields on the damage caused to lubricants.

Bliznyuk et al.^15^ compared the effect of ^60^Co γ radiation and reactor facilities on a commercial mineral oil (Leybonol LVO 500, Leybold, USA). Gamma irradiation was performed at a dose rate of 10–11 kGy/h, and the air temperature during irradiation reached 55 °C. The fast neutron flux corresponded to 1.8 · 10^12^ cm^–2^s^–1^. The oil samples irradiated in the reactor field showed a relative viscosity increase of 500% at about 0.68 MGy, and they became completely solid at 1.7 MGy. Relative viscosity increases of 200% and 500% were observed for the same oil irradiated with gamma sources up to 2 and 6 MGy, respectively. These results suggest a higher sensitivity to neutron radiation; however, the gamma dose component in this mixed field is unknown and was not considered. This leads to a dose underestimation possibly impacting the interpretation of these results and potentially contributing to the explanation of the observed differences.

Ferrari et al.^75^ reported the irradiation of MORESCO RP-42R oil in a mixed neutron and gamma field at a very high dose rate of 780 kGy/h at a temperature ranging from 50 to 70 °C, finding a viscosity increase of about 300% at 7.8 MGy. The viscosity-increase values for ^60^Co gamma irradiation at the same dose at a dose rate of 10 kGy/h and at room temperature were limited to about 30%,^60^ suggesting possible higher sensitivity to mixed-field doses. However, in addition to the different radiation fields, other parameters such as different dose rates could play a role.

## 6 State-of-the-Art Research Challenges

### 6.1 Inhomogeneous Irradiation Conditions among the Available Studies

**Figure 9:**
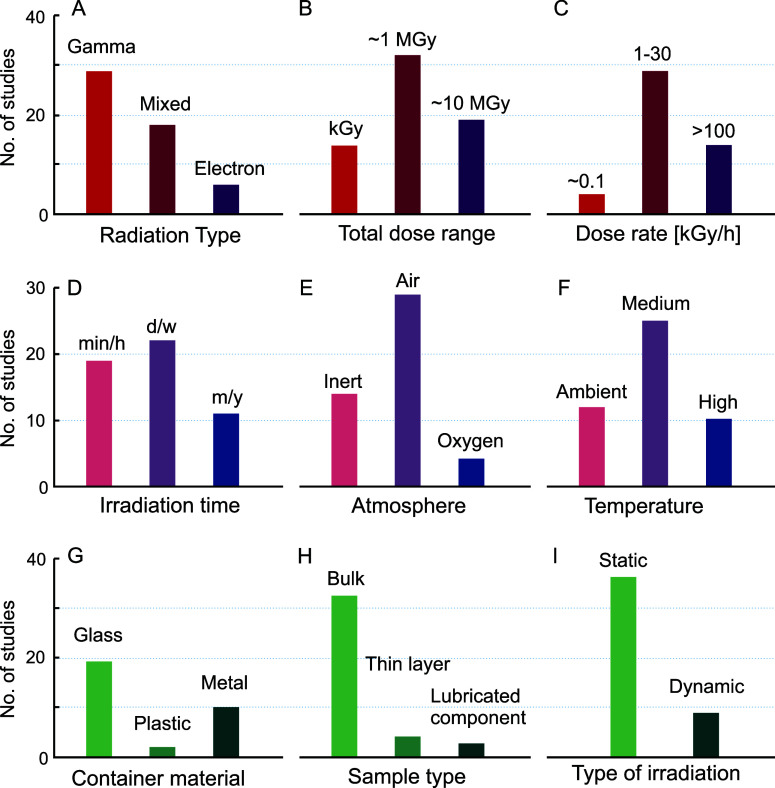
Number of studies for each type of irradiation conditions. Compiled examples of specific irradiation conditions reported in the literature^10−13,15,36,42,43,49,52,60,62,71−79,217,218,239−264^ were published after 1963 or were not included in the 1963 review by Bolt and Carroll.^3^ Typically, irradiation studies are performed with pure gamma sources (A) delivering ∼1 MGy (B) within a few weeks (D) at a dose rate of a few or tens of kGy/h (C). Bulk samples (H) contained in glass (G) are generally exposed in static conditions (I) to air (E) and an uncontrolled mild temperature rise related to deposition of energy from the radiation (F) that is usually difficult to quantify precisely. There is a clear under-representation of test conditions corresponding to real-life application conditions of lubricants in radiation areas, where most often thin layers within moving metallic components are exposed to low mixed dose rates over months or years. In addition, not all irradiation conditions were reported (or not with the same level of detail) in each analyzed study. Therefore, some parameters (e.g., container type) are underrepresented with fewer data points.

Despite the relatively small number of sources reporting new results on irradiated lubricants since the 1960s, the available data refer to inhomogeneous irradiation conditions and characterizations, making it extremely difficult to directly compare the available literature and organize the information into a coherent data set. The high variability between irradiation conditions and material formulations was previously reported in the 1980s.^66^ Examples of inhomogeneities within the irradiation conditions are presented in Figure 9. In addition to the comparability issue, most of the existing studies are difficult to apply directly to real-life applications since representative irradiation conditions are under-studied.

Precise monitoring or control of irradiation parameters is often not possible or limited by the constraints of the irradiation facility, both technical and related to the accessibility of irradiation slots. Consequently, parametric investigations are scarce, hindering a comprehensive understanding of the effects of radiation, temperature, and other physical stresses concurrently occurring in in-service conditions.^68,70^ For example, there is very limited knowledge of the effects of the dose rate and the combined effects of radiation and other stressors such as temperature.^68,70^

### 6.2 Inhomogeneous Post-Irradiation Characterization

The selection of materials for high-radiation areas is often based on the evolution of a certain material property with dose rather than the functionality of the lubricant in operation. In addition, postirradiation characterizations of lubricant samples reported in the literature are as varied as the selected irradiation conditions.

Typical quantities that are used to define the radiation stability and corresponding dose thresholds for organic lubricants include the worked and unworked consistency for greases^42,73,74,78,79^ and the kinematic viscosity at 40 °C and/or 100 °C for oils.^49,74,75,78,218^ Other common quantifications include analysis of the gases evolved during irradiation,^16^ drop point and oil separation,^15,73,74,78^ TAN,^3,72,74,75,77,218^ and color and texture analysis;^15,42,49,71,75,77,78^ structural and spectroscopic analyses include FTIR spectroscopy,^15,60,75,264^ nuclear magnetic resonance spectroscopy,^15,75^ gas chromatography,^16^ and gel permeation chromatography.^42,60^ G-value^72^ (where G is the radiolysis yield, defined as the number of molecules produced per 100 eV of energy absorbed) is a particularly interesting parameter, quantifying how many specific reactions (e.g., gas evolution) can occur in a polymer exposed to a given energy. Unfortunately, potentially the most interesting G-values for cross-linking and chain scission were not present in the reviewed studies of wet lubricants.

Macroscopic characterization can be combined with examination of optical properties such as radiophotoluminescence,^15^ and electrical properties.^60^ Radiation-induced color changes are often used to assess the amount of radiation damage visually and even quantitatively.^71,77^ Color changes may, however, not correlate with any other used end points.^265^

In some rare cases, functional parameters, such as wear,^67,218^ successful operation (as per its specification) of a lubricated component after irradiation,^10−13,76^ or lubricant loss from equipment,^49^ are evaluated in view of specific applications.

Possibly coexisting correlations between different markers of radiation tolerance are not obvious, leading to varying interpretations among authors. Moreover, the lack of standardization of the tested quantities and units used makes it challenging to interpret the available data. Consequently, more experimental results within the same category are needed to establish a consensus.

### 6.3 Definition of Usability Thresholds

Interpretation of data obtained from irradiation studies is another challenge for the selection of materials for radiation areas. In many cases, only qualitative assessments of the tested lubricants are conducted, making comparisons challenging. Moreover, when relevant parameters to be measured are defined, there is usually no consensus on specific thresholds, with users often defining their own criteria.

Historically, a conservative and simplified approach has involved identifying the most sensitive property to radiation among those tested, using it as an indicator of radiation tolerance.^66^ Dose thresholds were frequently associated with a 25% or 50% change in the reference property modified by radiation and the corresponding damage labeled as “moderate” or “severe,” respectively.^42,49,68^

Based on the considered quantities, ambiguities in the threshold definition can be found. For example, one possible definition uses the dose associated with viscosity change of +20% in bubbling oxygen (viscosity end point), and another by the dose corresponding to a TAN increase up to the reference value of 1.0 mg KOH/g (TAN end point).^77^

An estimate of the ratio between dose thresholds obtained using the two aforementioned approaches can be obtained by defining the usability-threshold ratio as follows:
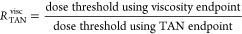


A systematic analysis of the data published by Nakanishi et al. on selected products allowed *R*_TAN_^visc^ to be calculated in the frame of the present work. It ranges between 0.8 and 2.5^77^ depending on the oil type. PPEs showed *R*_TAN_^visc^ values less than one due to critical TAN occurring at doses 20%–30% higher than the viscosity increase.^77^ Mineral oils, conversely, exceeded the TAN threshold sooner, resulting in *R*_TAN_^visc^ > 1.^77^

In practice, mechanical components may have a certain tolerance for degraded lubricants.^66^ Additionally, radiation might improve specific parameters while degrading others: for example, an improvement in wear life has been reported.^67,218^ As a result, static tests can be quite misleading for real applications,^66^ demonstrating the importance of evaluating lubricants in dynamic conditions^46^ and defining related thresholds.^218^

In summary, there is no universal agreement of general dose thresholds to assess the radiation resistance of lubricants, and this may pose challenges when selecting lubricants for actual use in high-radiation areas.

### 6.4 Radiation-Resistant System Design

**Figure 10:**
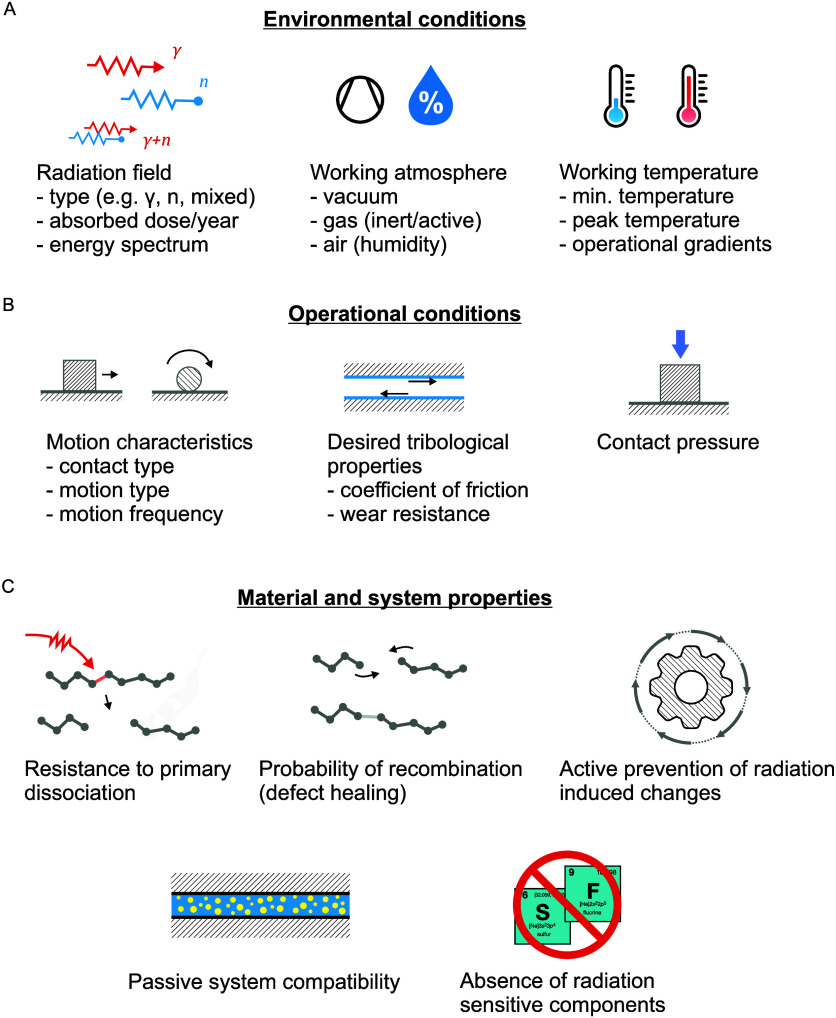
Radiation-resistant systems design. The design of a radiation-resistant system can be achieved through systematic analysis of (A) specific environmental conditions, such as the type of radiation field, working atmosphere, and working temperature, as well as (B) operational parameters such as motion characteristics, target tribological properties, and contact pressure. (C) Knowing the conditions, one can appropriately design the system and materials to achieve reliable and safe operation.

The design of radiation-resistant lubricated equipment requires considerations of the environmental conditions, of the operation, and of the properties of the used systems and materials, as shown in Figure 10. It is important to note that lubricants are useful beyond their tribological properties. Some applications, such as oil diffusion pumps or corrosion protection coatings, utilize different material properties of lubricants. All, however, can be subjected to radiation-induced damage and potential failure. In this context, all failure risks (and all desired material properties) should be defined individually for each specific application.

Different strategies can be adopted, including anticipating radiation-induced system modifications and evaluating possible compatibility of the system with these expected changes. Lubricants should be avoided when feasible, and maintenance of lubricated components should be analyzed.

Some types of radiation damage in lubricants can be actively prevented or delayed by using specific components or procedures. For example, radical scavengers are able to terminate oxidation chain reactions.^3^ Mechanical motion has been reported to delay gelation/grease hardening,^103^ so the regular movement of lubricated mechanisms is beneficial not only to redistribute the lubricant but also to decrease the rate of cross-linking. Some radiation-induced effects can also be partially reversed by annealing.^3,61,86−91^

Some changes affecting a specific property of the irradiated material can partially offset each other. For instance, the thickening of the base oil in grease, which increases the overall hardness of the lubricant, can be counterbalanced by the breakdown of the thickener structure, which softens the lubricant.^42,103^ Other radiation-induced effects may be irrelevant for a specific operation, such as changes to the melting point if the system does not operate at high temperatures. In other cases, the design of the system can be adapted to anticipate or mitigate certain effects of radiation—typically those that are most likely to occur or are the most severe—without compromising overall functionality.^25,266^ One limitation of this approach is that it relies on the assumption that the radiation damage observed during testing—which, as noted, is typically performed in an accelerated way and under standard irradiation conditions—will occur in a similar way during operation. The validity of this approach will be further discussed in Section 6.5. This highlights the importance of considering lubricant selection for radiation-prone areas during the design phase.

The radiation resistance of a lubricated system can be increased by avoiding the most sensitive materials or by locating them as far as possible from radiation sources. For example, as noted in Section 5.1, fluorinated (and more generally, halogenated) products typically exhibit increased radiation sensitivity;^3^ in fact, they tend to form large amounts of corrosive acids in the presence of oxygen, even at low radiation levels,^60^ and this could impact the operation and safety of the equipment of which they are part. Lubricants containing silicone oils or phosphates should also be avoided in high-radiation areas, as discussed earlier.^3,60,219^

When using lubricants in high-radiation areas is considered unavoidable, frequent preventive maintenance or replacement of the lubricated components should be planned to minimize the risk of equipment damage and possible failure. However, high-radiation areas are usually associated with access constraints, in most cases limiting maintenance and human access.^25^

**Figure 11:**
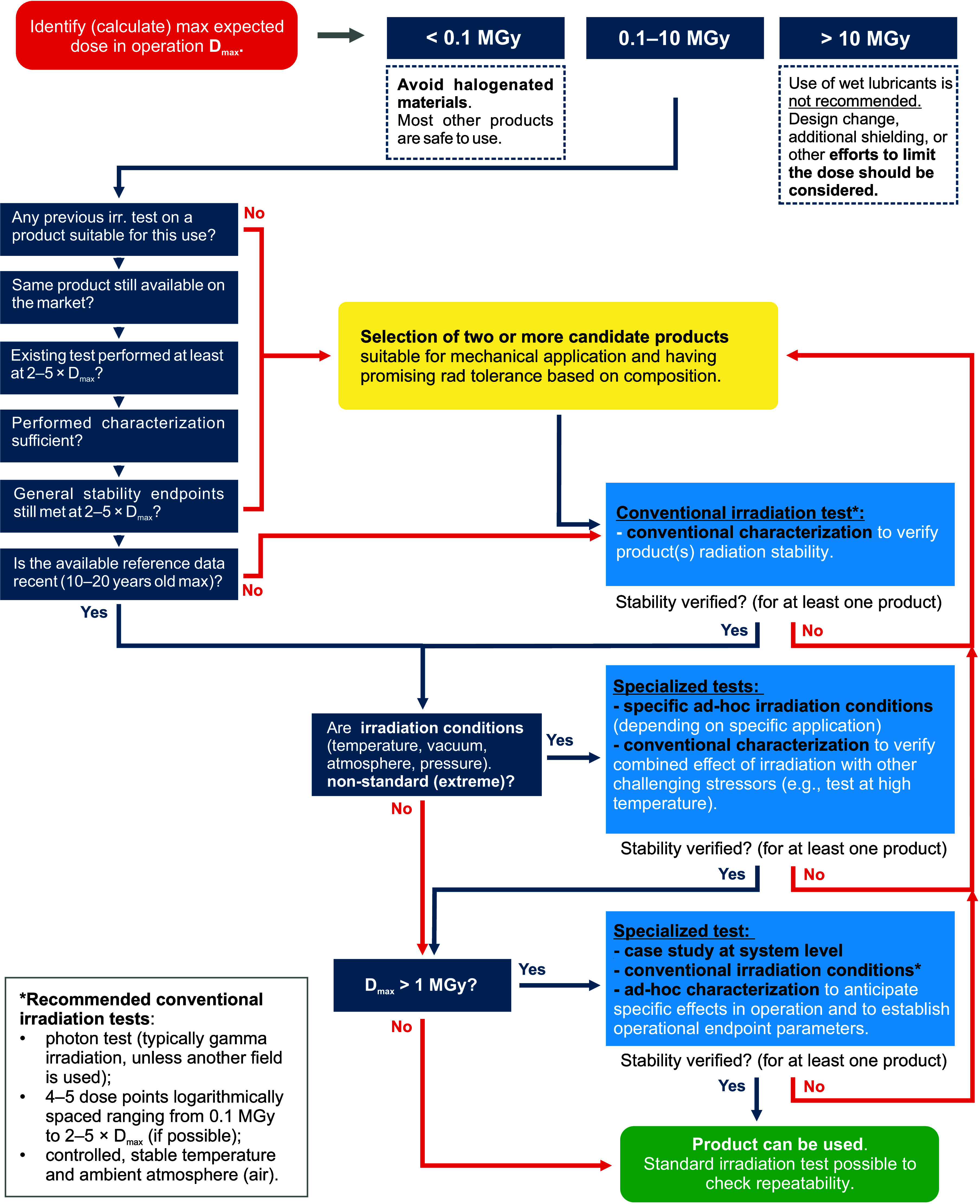
Decision tree of general guidelines for selecting lubricant materials for radiation environments based on the maximum expected absorbed dose in operation, *D*_max_. These recommendations focus solely on radiation-related aspects; other technical constraints should be addressed separately.

These general aspects provide a foundation for estimating the expected radiation resistance and thus serve as a preliminary guide for selecting lubricant candidates. However, specific studies on the materials of interest are essential before finalizing lubricants for critical applications in high-radiation environments, as discussed in Section 7 and shown in Figure 11.

### 6.5 Lack and Incompleteness of Open Experimental Data

A large portion of the literature on radiation damage remains inaccessible to the public due to proprietary restrictions, limiting the availability of valuable information.^66^ Historical challenges in data collection and accessibility have necessitated specific agreements with institutions, such as the European Space Agency, to grant access to valuable data.^267^ Furthermore, producers and institutions conducting radiation-damage tests are often not interested in making their results and methodologies public.^66^

The absence of accessible, comprehensive, and integral data sets poses significant challenges for designers of equipment for radiation areas,^69^ who often have to rely on previous tests that may not be sufficiently representative of the expected operation conditions.^70,75^ Moreover, as most available data were collected decades ago, it is often impossible to contact the authors of the relevant studies to gather additional information on the products tested and the irradiation and testing methodologies. For example, despite decades-long efforts by CERN to compile information on materials within the Yellow Reports, the gathered data are reported to be incomplete (and presently outdated since some tested products do not exist on the market anymore), indicating the need for further investigations.^49,70^ Specifically, there is a dearth of data collected at radiation doses exceeding 10 MGy, which is crucial for various extreme operation conditions.^267^

Additionally, many commercial products tested over recent decades are now obsolete, out of stock or production, or generally unavailable. Even when radiation tests were conducted on specific products, these may be unusable if the product in question is no longer available or its specific formulation has significantly changed. Indeed, radiation tolerance is not typically a primary requirement for lubricant performance and may not be preserved in composition upgrades. Large sensitivity variations between supposedly similar materials from different manufacturers also contribute to the lack of reliable references, demonstrating the need for collaborations with manufacturers to successfully complete radiation-damage studies.^42,75,267^

The general lack of data has led to decades of speculation based on the limited experimental results available, with little progress in advancing actual knowledge on the topic. This has allowed unverified hypotheses to become established and culturally accepted, despite the absence of thorough and systematic validation.

Although accelerated testing remains a powerful and valuable tool for equipment qualification, identifying the most likely dominant failure modes in comparative materials evaluations and determining component vulnerability to aging processes, it may have a limited relationship with the actual damage experienced in operation.^68,71^ The conditions under which extrapolations to service life are made are not always clearly understood or scientifically rigorous. Moreover, implementing accelerated tests to predict the effects of radiation remains extremely complicated.^100^

Finally, follow-up studies considering the evolution of lubricants in high-radiation areas during and after their use, along with the regular and systematic reporting of failures, are largely missing from the literature. Such analysis is crucial for optimizing material development and design choices.^16,47^

In light of these challenges, potential strategies for mitigation include collaborative efforts between institutions, the comparison and possible harmonization of existing databases,^267^ and the establishment of standardized open-access repositories for data and methodologies.

### 6.6 The Need for New Data on Emerging Lubricant Materials

The need to expand and enhance the knowledge related to the effects of radiation on materials and accelerated testing processes has been repeatedly reported since the 1960s to the present day.^3,42,43,49,66,68−70,268,269^ Aside from being crucial for radiation areas, the need for optimized material solutions and extended equipment lifetimes represents a significant societal and ecological necessity.^47^ In addition to the scientific literature, several recent press releases emphasize the need to conduct materials studies—particularly on radiation-resistant lubricants—for the development of key future technologies, underscoring the resurging importance of these niche yet fundamental scientific investigations^270,271^

Access to irradiation facilities capable of delivering doses in the MGy range to macroscopic material samples within timelines compatible with design schedules presents a significant challenge in collecting new data. In fact, these data are challenging to produce in terms of facility availability and costs. Creating a network of facilities and laboratories capable of irradiating and characterizing materials up to the MGy dose range and allowing systematic parametric investigations would significantly expand the available data set.

### 6.7 Dosimetry

There has been a long-standing debate regarding the impact of radiation type and energy spectrum on the overall effects of radiation on organic materials. Various authors believe that the radiation-induced effects ultimately depend on the total dose absorbed by the material;^48,49,66^ others limit the validity of this approximation only to cases in which irradiation is performed under similar circumstances.^69^ Caution is generally recommended when there are large differences in particle type and energy spectrum, temperature, oxygen concentration, or dose rate.^69^ This topic has been controversial due to the lack of evidence.^42,100^

Differences between the effects of γ radiation and mixed reactor radiation on dry-film lubricants were reported by Cosgrove and Dueltgen;^218^ additionally, neutrons have been reported to be more damaging to organic lubricants than gamma sources.^15,75,77^ Complete dosimetry information is, however, missing in most cases, as is an understanding of the influence of other specific factors.^100^ It is, in fact, technically challenging to deliver equal doses in different radiation environments while keeping other irradiation parameters (such as dose rate, temperature, and atmosphere) unvaried. Next-generation neutron-dominated irradiation stations^52^ allow doses in the MGy range to be delivered to materials using a low dose rate (about 0.1 kGy/h). These irradiation conditions could be matched with standard gamma-irradiation conditions to verify the discussed assumptions. Nonetheless, as it stands, there is insufficient data to reach a general consensus on this point.

An additional problem is that dosimetry often lacks detailed and specific considerations. For example, in the Yellow Reports,^48^ doses are reported in Gy for a generic organic (CH_2_)_n_-based material, irrespective of the actual composition differences, which can impact the total absorbed dose.^42,48,49^ Indeed, in many cases, the facilities in which irradiation is performed provide dosimetry references based on the industrial standards used for calibrating the facility.^15^ These results are often not specific to the irradiated materials. Precise dose assessments typically require a detailed knowledge of the used irradiation field and irradiation parameters. A combination of experimental dosimetry and Monte Carlo simulations to model the transport and interaction of the radiation fields in the irradiated samples is generally necessary to calculate the dose actually absorbed by the irradiated sample.^42,52,75^ This information is generally not reported or, when available, it is incomplete and based on assumptions that do not allow the true absorbed dose to be estimated *a posteriori*, and prevent effective comparisons between different irradiation studies from being made. In this manuscript, the dose values reported in the original papers are used. Monte Carlo simulation tools can complement experimental tests in studying the evolution of materials. However, simulations cannot yet predict the expected dominant effects; in these models, the molecular structure of the material is not represented, and radiation-damage mechanisms are not fully understood, making experimental tests fundamental for advancing knowledge.

Nevertheless, in most radiation studies on materials, despite the lack of detail on how it is estimated, the total integrated physical absorbed dose is the quantity to which the effects of radiation and damage levels are referenced.^3,11,15,42,43,49,52,60,66−75^ Considering the limited knowledge about the effects of radiation on complex materials and the lack of well-understood connections between phenomena occurring at different scales—such as the physical, chemical, structural, mechanical, and functional scales—the use of the total dose (which is by definition an averaged quantity) as the only relevant physical parameter to which damage should be referenced could be questioned.

Finally, the irradiation of materials in neutron or heavy, charged particle fields might lead to the activation of specific atomic species present in the material composition, making them radioactive. For organic lubricants, this typically concerns the metallic impurities contained in the commercial product.^30^ Activated materials might fall in the legal definition of radioactive items, additionally complicating their handling, shipping, storage and final disposal. Despite the relevance in the radiation protection point of view, the dose contribution deposited in the sample due to this activity is usually several orders of magnitude lower than the dose absorbed during irradiation, and as a first approximation, it can be neglected in testing conditions.^30,52^ However, in operation conditions, the residual activation not only of the lubricant but of the whole equipment can lead to additional gamma dose contributions that, if integrated over long postirradiation times, can be nonnegligible and need to be carefully estimated.^55^

## 7 Outlook

With the rise of increasingly complex systems and projects designed to operate in high-radiation environments, the development of radiation-resistant materials is crucial for secure, reliable, and optimized operations. A comprehensive study approach is essential for developing, understanding, and applying both conventional and emerging materials to enhance the performance of lubricants in radiation environments and to address the uncertainties present in this field. Specifically, parametric studies to systematically examine and clarify the dependence of radiation damage on specific parameters are urgently needed.

An important aspect of radiation studies of materials is the use of a multiscale approach, which involves simultaneously investigating radiation damage across nano-, micro-, and macroscales. Such an approach would allow us to deepen our understanding of physical phenomena and their direct impact on the functional properties of a system. New prospective research facilities, such as CERN’s Beam Dump Facility and the Search for Hidden Particles^272^ experiment, could accommodate a dedicated materials-irradiation station and facilitate operation-representative studies of new materials for future applications such as the CERN’s Future Circular Collider.^273,274^ A shift in approach is needed to enhance the knowledge base and ensure comparable studies and safe operations in various engineering fields exposed to high-radiation environments. Actions are required from all stakeholders, including researchers, research institutions, irradiation facilities, and lubricant manufacturers. Table 2 provides proposed long-term strategies and actions for systematically tackling the investigation of the effects of radiation on lubricants for present and future needs, listing those who need to be involved in the implementation of each.

**Table 2:** Long-Term Strategies for Enhancing the Understanding of the Effects of Radiation on Lubricating Materials and Their Practical Implementation

**Actions**	**Actors**
Formulate general guidelines for the irradiation and postirradiation characterization of lubricants in terms of target dose, dose rate, environmental irradiation conditions, amount of material to be tested, irradiation containers, and setups. Identify a generally agreed set of quantities to be tested and allow variations for defining common and comparable thresholds.	Researchers.
Establish and maintain a collaborative network of universities and institutions dedicated to sustaining long-term studies of radiation damage.	Institutions.
Form partnerships with manufacturers to gain insights into lubricants, characterize specific commercial formulations, and potentially design radiation-tolerant products.	Institutions and lubricant producers.
Secure access to a network of irradiation facilities capable of and willing to perform systematic and parametric radiation-damage studies.	Institutions.
Standardize experimental dosimetry procedures to estimate the total dose absorbed by irradiated lubricant samples.	Researchers and irradiation facilities.
Follow up on the actual use of lubricants in high-radiation areas, noting design choices and reporting failures.	Equipment owners and researchers.
Implement and develop simulation methodologies to model and anticipate radiation effects in lubricating materials (existing and to be designed) and lubricated components in operation.	Researchers and institutions
Produce and update roadmaps to anticipate future needs, constraints, and risks associated with using lubricants in high-radiation areas.	Institutions.
Include lubrication considerations in the early design phases of equipment intended for operation in high-radiation conditions.	Designers and equipment owners.
Ensure that collected data are published in peer-reviewed open-access journals or made available in open archives.	Researchers, producers, institutions, and irradiation facilities.
Establish adequate discussion spaces on this subject at conferences and workshops, with dedicated sessions, and ensure dissemination to the general public.	Institutions.
Develop and apply machine-learning methods in the design of radiation resistant materials (lubricants).	Researchers and institutions.

The current state of the art in radiation-tolerant lubricants presents both challenges and opportunities. Emerging materials hold promise as candidates for highly efficient lubrication and surface protection, provided they are properly tested and designed. Nanomaterials have the potential to reduce friction and wear in both dry and wet lubricating conditions; as lubricant additives, they can improve lubrication, particularly under dynamic, variable work conditions or when changes in oil viscosity are anticipated throughout the operational lifetime.^145^ Depending on their structure, size, and chemistry, several speculative lubrication mechanisms for nanomaterials have been proposed in the literature,^2,275^ and these can be expanded by exploiting abundant chemistry and potential chemical modifications to tune specific parameters of the material (see Section S4 and Figure S3 of the Supporting Information). Throughout recent academic and industrial studies, nanoparticle-enhanced lubricants have proven to perform well and satisfy the needs of most extreme work environments,^187,208,276,277^ potentially including high-radiation applications.

Including nanomaterial additives in conventional wet lubricants is not necessarily intended to enhance wear and friction properties but rather to extend the functional lifetime of the lubricant. With the susceptibility of organic materials to radiation damage, lubricious particles may enable effective reductions of wear and friction when an organic oil matrix fails due to radiation-induced decomposition. Nanomaterials could also be employed to improve the radiation resistance of products already considered radiation tolerant. The added benefit of enhancing tribological properties and enabling self-repair makes nanoparticle-infused lubricants potentially attractive future materials for the most extreme of radioactive environments.

Another challenge is the lack of a clear pathway to design, select, and validate materials for specific high-radiation applications. Given the complexity of radiation environments and the fragmented current state of the art, we propose a set of general guidelines (in the form of a decision tree) that can be applied to selecting appropriate lubricant materials for specific radiation conditions (Figure 11). Due to particular hazards in radiation environments, these guidelines should be considered when relubrication, (preventive) maintenance, or replacement of lubricated equipment is not possible or practical.

## 8 Conclusion

Lubricating materials are crucial to ensuring safe and efficient operation of mechanical components. In radiation environments where maintenance access is limited and machine failure could lead to dangerous and costly incidents, the proper selection and use of appropriate lubricants are paramount. Unfortunately, the current understanding of radiation damage in lubricants is often based on old, outdated experimental results and anecdotal evidence. The rapid development of the nuclear, fusion, aerospace, medical, and particle-research sectors will inevitably give increasing prominence to the significant shortage of usable data and systematic research practices. Given the abundant variety of available lubricants, both wet and dry, and current research trends in the field of tribology, radiation-resistant lubricants must be systematically and thoroughly investigated to ensure consistent and comparable results. Moreover, to ensure the reliability of experimental results and, ultimately, of lubricated equipment, one must consider the engineering requirements along with the specifics of the irradiation conditions, radiation type, dosimetry, and environmental factors. A multiscale approach to experiment design will offer a comprehensive solution, enabling an understanding of material behavior across the nano-, micro-, macro-, and operational-system scales.

Ionizing radiation fields often have unintuitive and unexplored effects on materials and their macroscopic properties. The mutual influences of additives and their radiolytic products may lead to phenomena such as self-healing, radiation-induced improvements in properties, or unexpected radiation tolerance or sensitivity. These challenges offer intriguing opportunities for study, with the potential to yield surprising results and develop new materials tailored for specific engineering problems in radiation environments. There is plenty of room to explore the richness of organic chemistry, the complexity of nanostructures, and the mutual and intricate influences of various additives in radiation environments.

The design of radiation-tolerant materials is a broad and multidisciplinary field, and it requires a deep understanding of chemistry, radiation, materials, dosimetry, and engineering. The growing demands of research and industry are increasingly placing pressure on the academic community to investigate and identify radiation-tolerant materials. The ever-greater attention being paid to sustainability also highlights the need to optimize the lifetimes of materials and equipment while minimizing environmental impacts, resource consumption, and waste. Lubricated mechanisms are complex systems in which the fields of chemistry, materials science and mechanics interlace and are further complicated by the presence of radiation. As such, they require a proactive research approach and a gentle shift in the planning of radiation-damage studies. Soon, technological developments in the fission, fusion, medicine, space, aeronautics, particle-research, and irradiation sectors will outgrow the capabilities of conventionally used materials. This, however, creates an exciting opportunity to push the boundaries of the design of radiation-tolerant materials, deepening our understanding of the complex effects of radiation damage and ensuring machine and operator safety, energy efficiency, and durability in high-radiation environments.

## Supporting Information Available

Historical overview; the role of lubricants in mechanical contacts; effects of radiation damage on polymer molecules; radiation tolerance data for experimentally tested oil and grease products; types of nanostructure used as lubricant additives; mechanisms associated with nanoparticle lubricant additives (PDF)
