# Supplementary material for: Effects of Radiation Dose on Lubricants: A Review of Experimental Studies
Source: ACS Appl Mater Interfaces. 2025 Feb 27;17(10):14773–800. doi: 10.1021/acsami.4c21220 (PMC11912217; doi:10.1021/acsami.4c21220)
Supplement: Supplementary file 1 — am4c21220_si_001.pdf [file am4c21220_si_001.pdf]

Supporting Information: Effects of Radiation Dose on Lubricants: A Review of Experimental Studies

Michal Macha,[*,†,‖] Dominika Senajova,[†,‡,‖] Tim Giles,[†] Marco Calviani,[†] Sylvain Girard,[¶,§] and Matteo Ferrari[*,¶,‖]

†*CERN, CH-1211, Geneva 23, Switzerland*

‡*Imperial College London, SW7 2AZ, London, United Kingdom*

¶*Université Jean Monnet, Saint Etienne, CNRS, Institut d'optique Graduate School, Laboratoire Hubert Curien UMR 5516, F-42023, Saint-Etienne, France*

§*Institut Universitaire de France (IUF) Ministère de l'Enseignement Supérieur et de la Recherche 1 rue Descartes 75005 Paris, France*

‖*These authors contributed equally to this work.*

*E-mail: michal.macha@cern.ch; matteo.ferrari@univ-st-etienne.fr

List of Figures

S1 Radiation tolerance of oil products based on their base-fluid composition. . . 7

S2 Radiation tolerance of grease products categorized by their oil base. 8

S3 Mechanisms associated with nanoparticle lubricant additives. 10

S1 Historical Overview

The investigation into the radiation resistance of lubricants dates back to the 1950s and 1960s, coinciding with the development of nuclear technology and its applications in civil and military sectors, including power plants, nuclear-powered aircraft, naval vessels, rockets, and turbines. The general vulnerability of lubricants to radiation damage has already been established, meaning that it is vital to explore the effects of radiation on their properties. Early studies and patents focused on the development of radiation-resistant lubricants for these emerging nuclear applications.[S1–S6] The extreme irradiation conditions in these environments included intense mixed neutron and gamma radiation fields, high temperatures, and integrated doses reaching tens of MGy.[S3,S7] In parallel, the progress in particle-accelerator technology motivated irradiation studies of commercial oils and greases in mixed radiation fields and at room temperature.[S8]

In the 1970s, NASA investigated the radiation resistance of structural materials for space missions using reactor radiation.[S9] Guidelines for the selection of materials for missions to Jupiter were published during this period, and these specifically considered proton and electron damage.[S10] A few other studies reported results for lubricating oils irradiated with ^{60}Co and/or reactor sources.[S11–S13]

The 1980s brought a new wave of interest in radiation-resistant materials, possibly motivated by research institutions. The Georgia Institute of Technology conducted a comprehensive literature review on the effects of radiation on organic materials, including lubricants, focusing on nuclear power plants.[S14] This review aimed to identify safe dose thresholds for the use of these materials. Despite the limited amount of original data on lubricants from the 1960s to the 1980s, the review emphasized the ongoing research efforts at institutions such as Sandia Laboratories and CERN in gathering data on the performance of materials in radiation environments.[S14] CERN, in particular, conducted extensive irradiation campaigns to test commercial components for use in high-energy particle accelerators.[S8,S15,S16]

A comparison of the effects of radiation on several commercial lubricating oils was

published in 1984, evaluating the impact of very high doses (up to 10 MGy) under different irradiation conditions, such as vacuum and oxygen environments. This study examined changes in viscosity and total acid number (TAN) with respect to absorbed dose.[S17] Around the same time, Japanese companies, in collaboration with the Japanese Atomic Energy Agency, developed and patented formulations for radiation-resistant commercial lubricants to meet the demands of high-radiation applications in nuclear environments.[S18] Following the recommendations from previously published literature, polyphenyl ethers were selected as the basis for these formulations.[S3] As expected, in a systematic and extensive irradiation study of lubricating oils,[S19] these special formulations outperformed generic products; they demonstrated limited changes in viscosity, TAN, and infrared spectra, along with low gas evolution.

Continued efforts to improve the radiation resistance of lubricants were seen in patents and studies from the 1980s, during which time the focus shifted from oil formulations to the development of greases capable of maintaining radiation tolerance even under high-temperature conditions.[S20–S22] These products still represent the most radiation-resistant options available today, and the authors of these works have reported no significant further advancements in the commercial production of other equally resistant radiation lubricants.

Only a few recent publications have reported the investigation of multiple commercial products,[S23] various irradiation conditions, or the evolution of a variety of lubricant properties.[S24,S25] In fact, recent decades have been dominated by case studies on the selection of materials, including lubricants, for specific applications, spanning fusion,[S26–S31] particle accelerators and accelerator-driven scientific facilities,[S32–S37] commercial irradiation facilities,[S38] space applications,[S25] and the decommissioning of nuclear infrastructure.[S39]

Given the criticality of these fields and their continuous development, it can be assumed that an increasing number of mechanical components exposed to increasingly high doses of radiation will need reliable lubrication. In addition, the dose levels typically encountered in operation might be beyond the stability thresholds of conventional and even current

radiation-tolerant lubricants.

Despite the quality and breadth of previously published studies and reviews, they may not be practical for design engineers for one or more of the following reasons:

- early studies exist only in paper format or as scanned documents, and they might not be automatically searchable;
- previous results are highly inhomogeneous and often reported in different units across countries and decades (e.g., for dose, temperature, lubricant properties, etc.), so comparisons might require unit conversions;
- reports might concern pure compounds or discontinued commercial products, so a good knowledge of the lubricant market and compositions is needed to identify similar products;
- most reports concern a wide number of different materials, so information specifically relating to lubricants is scattered across numerous publications;
- not all research cited in this field is publicly available, and it often requires paid access to scientific journals.

As a result, the present review is believed to be a useful reference for selecting lubricants for areas exposed to radiation. The identification and selection of materials with enhanced radiation properties are crucial for minimizing failure risks and, consequently, reducing health impacts (such the exposure of operators to radiation), costs, environmental effects, and the likelihood of accident scenarios.

S2 The Role of Lubricants in Mechanical Contacts

The primary function of lubricants is to reduce friction between moving parts in contact by forming a thin, low-shear layer between surface asperities. Such an intermediate film reduces

the effective contact area, protects the material from wear and/or oxygen access (reducing potential oxidative corrosion), and enables smoother and safer operation.[S40] Such a low-shear film can be provided either by a fluid (wet) lubricant or by lamellar solids.

S2.1 Composition and formulation

In the present work, the term "wet lubricants" is used to describe water- and oil-based fluids, waxes, and greases. Due to issues related to corrosion and load-carrying capacity, water-based fluids are not deemed suitable for radiation applications and will not be discussed in detail here. Lubricating oils usually contain a synthetic or mineral-oil-derived base stock and a set of oil-soluble additives to provide the desired properties and resistance to environmental conditions. Mineral-oil-based fluids usually contain a number of hydrocarbon species and trace compounds,[S41] while the composition of synthetic oils is better defined.[S42] Waxes are composed of long aliphatic (i.e., not aromatic) hydrocarbons with melting points above room temperature.

S2.2 Relevant Parameters and Measured Quantities

The motion of loaded surfaces is resisted by a friction force that is proportional to both the normal load and the coefficient of friction (CoF or μ). The presence of lubricants enables the CoF in dry metal-on-metal contacts to be reduced from $\sim$0.5 to $\sim$0.1 or even lower. In the case of fluid lubricants, the CoF is related to viscosity and speed of motion according to the so-called Stribeck curve.[S43] The viscosity of a fluid quantifies its resistance to flow; both kinematic (ν measured in $\mathrm{cSt} = \mathrm{mm}^2 \cdot \mathrm{s}^{-1}$) and dynamic ($\eta$ measured in $\mathrm{mPa} \cdot \mathrm{s}$) viscosity are widely used, and these are related via the fluid density ρ according to $\eta = \rho \times \nu$.

Grease consistency grades are defined by the National Lubricating Grease Institute according to the results of worked cone penetration tests. Results are obtained by measuring the penetration depth of a standardized cone during a 5-s free fall in a defined geometry. Before the test, grease is typically worked using a dedicated handler, which forces the

grease to pass repeatedly through holes of a specific size. The cone penetration test provides information about changes within the grease structure, and its results are somewhat correlated with grease rheology;[S44] however, their relationship with tribological performance (e.g., CoF) is not uniform across different grease types.[S45] More detailed and complementary information about lubricant structure can be obtained using gas permeation chromatography (measuring the lubricant's molecular-weight distribution), scanning electron microscopy (visualizing the lubricant's structure), or rheology (quantifying the lubricant's viscoelastic properties). Chemical changes can be highlighted with techniques such as Fourier transform infrared spectroscopy, Raman spectroscopy, nuclear magnetic resonance spectroscopy, or X-ray photoelectron spectroscopy.

TAN, measured in mg KOH/g, quantifies the content of acid species by measuring the base needed to neutralize them. Some acids can promote the corrosion of metals, and lubricants should therefore be replaced before a critical value of TAN is reached. However, the exact value of this critical threshold varies from 0.3[S42] to 3.0 mg KOH/g.[S46] In addition, the presence of certain types of additives can lead to non-zero TAN values even in fresh lubricants.

S3 Radiation Tolerance Data for Experimentally Tested Oil and Grease Products

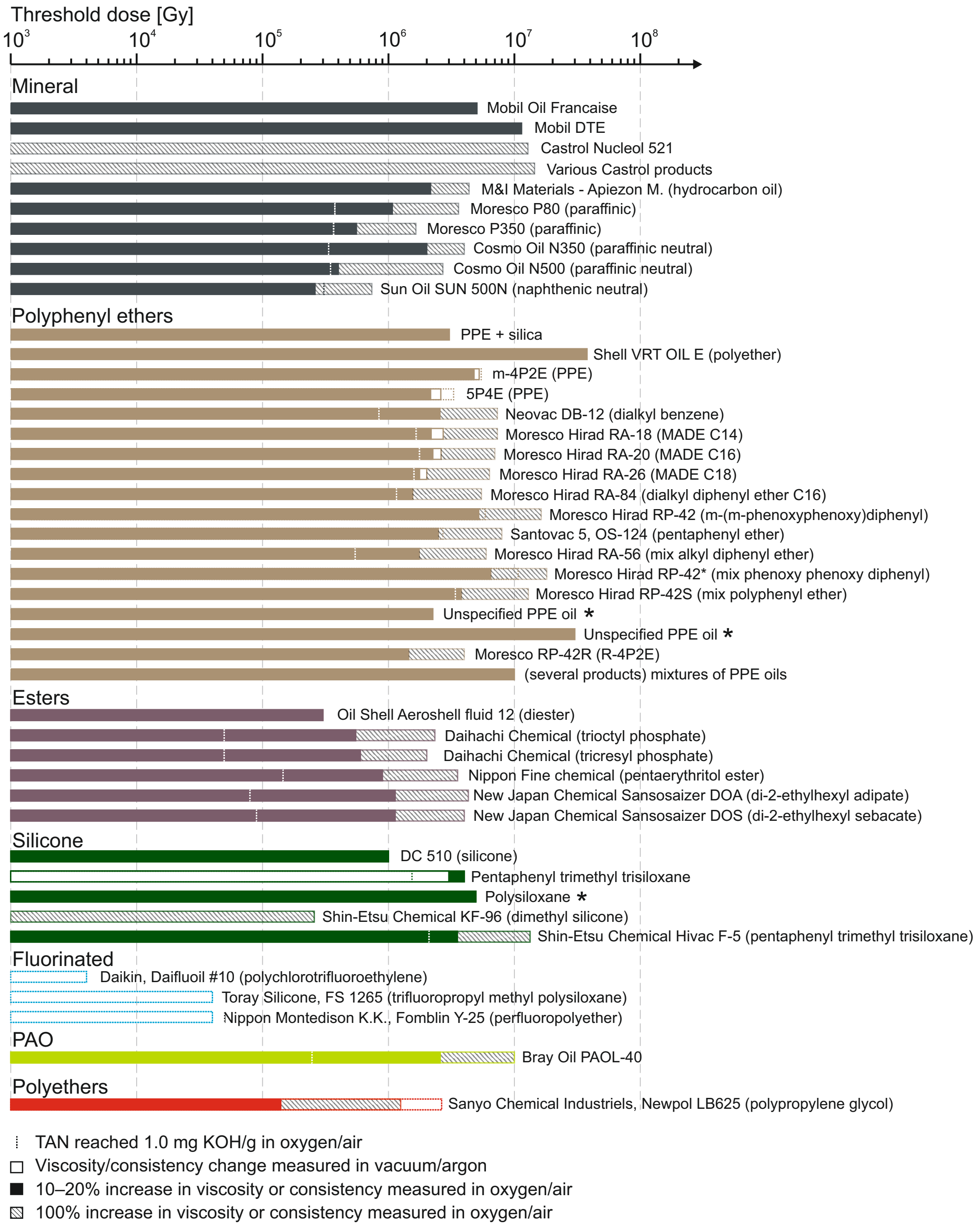

Figure S1. Detailed comparison of available experimental data on radiation dose thresholds for oils based on their measured viscosity increase and observed TAN measured in air or oxygen. This compilation is based on published radiation-test data on mineral,[S8,S19,S31,S47] polyphenyl ether,[S8,S17,S19,S20,S22–S24,S29,S48,S49] ester,[S5,S19] silicone,[S8,S17,S19,S29] fluorinated,[S19] polyalphaolefin (PAO)[S19] and polyether-based oils.[S19]

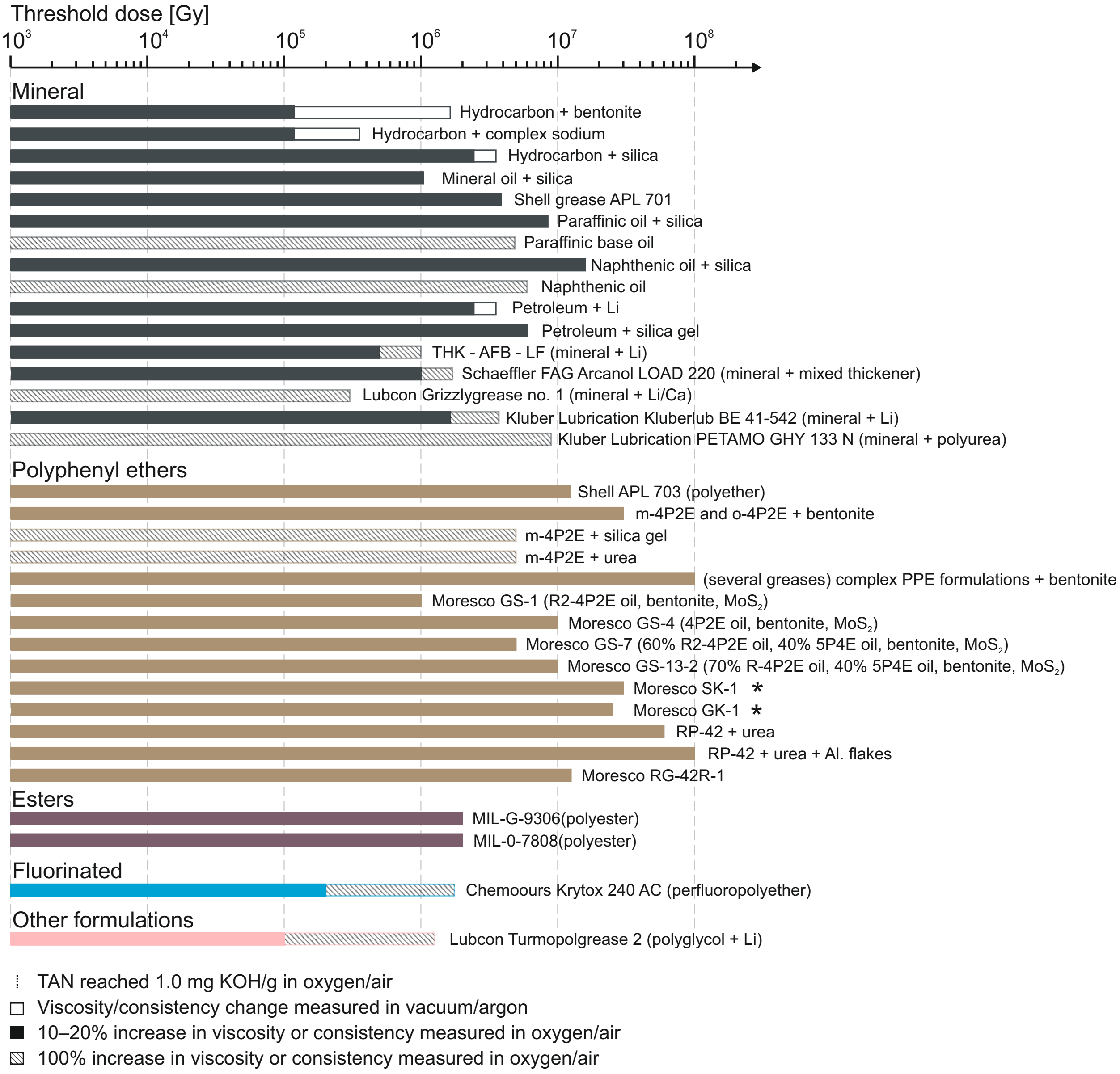

Figure S2. Detailed comparison of available experimental data on radiation dose thresholds of greases based on the consistency-change endpoints. Data were compiled based on published results of radiation tests on mineral,[S3,S20–S24,S50] polyphenyl-ether-[S8,S18,S21,S22,S27,S28,S30,S51] ester-,[S8] and fluorinated-oil-based greases,[S23,S24] along with other unspecified formulations.[S23,S24]

S4 Types of Nanostructure used as Lubricant Additives

Unlike their application as solid, dry film lubricants, nanomaterials for liquid lubrication primarily involve the use of nanoparticles. The literature typically classifies these materials based on their nanometric dimensionality: zero-dimensional (0D) nanostructures, such as nanodiamonds, fullerenes, and quantum dots; one-dimensional (1D) structures such as nanotubes and nanowires; and two-dimensional (2D) materials, including laminar substances such as graphene, hexagonal boron nitride (hBN), and molybdenum disulfide (MoS_2).[S52] Currently, the most studied lubricious particles relevant to radiation environments include carbon-based materials (such as graphene derivatives), MoS_2, and boron nitride nanostructures and allotropes.[S53–S57]

S4.1 Lubrication Mechanisms

Generally, the primary reason for the enhancement of wear properties with nanoparticle additives is their nanoscale size. Such small particles can enter the narrow contact area and directly affect the contact conditions between the working surfaces through friction and wear reduction.[S57] Several mechanisms have been proposed to explain this phenomenon in detail. Depending on their structure and size (Figure S3A), nanoparticles suspended in oil can act as shearing films[S58,S59] or nanoscale ball bearings,[S60,S61] potentially regulating the lubricant flow and reducing friction losses.[S57,S62,S63] Due to their weak interlayer interactions, nanostructures can promote the formation of tribofilms[S64–S66] and intermix with nanocrystalline or amorphous areas of the substrate, enabling a wear-free sliding regime[S62,S67] or filling/mending rough surfaces.[S58,S68,S69] Some types of nanoparticle are also associated with asperity-reducing nanoscale-polishing effects,[S61,S70] surface passivation,[S71,S72] or corrosion-inhibition effects[S61,S70] (Figure S3B).

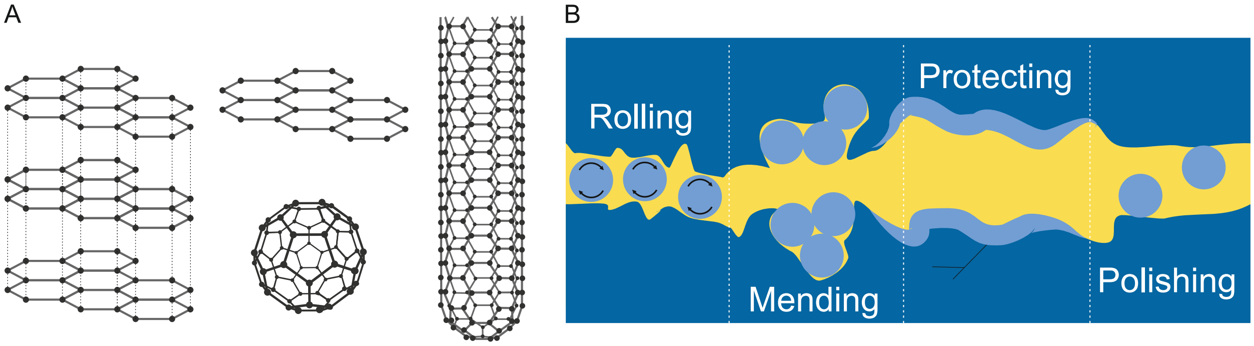

Figure S3. Schematics illustrating the lubrication mechanisms that dominate with the application of different kinds of nanoparticle lubricant materials. Depending on their structural shape (A), nanoparticles are hypothesized to improve the system through specific lubrication mechanisms occurring at the nanoscale (B).[S57]

S4.2 Functionalization

Despite their nanoscale dimensions, which should theoretically allow for Brownian motion dispersion,[S73] nanoparticles have a strong tendency to agglomerate. Therefore, a functionalization step prior to mixing is essential to achieving a stable and homogeneous dispersion in the lubricant fluid.[S57]

References

(S1) Wilson, E. V. Lubricants Resistant to Atomic Radiation, US Patent 3028334. 1962.

(S2) Rice, W. L. R.; Cox, W. L. *The Effects of Nuclear Radiation on Solid Film Lubricants, WADC-TR-58-499*; 1959.

(S3) Bolt, R.; Carroll, J. *Radiation Effects on Organic Materials*; Academic Press: New York, 1963.

(S4) Cosgrove, S. L.; Dueltgen, R. L. *The Effect of Nuclear Radiation on Lubricants and Hydraulic Fluids*; 1961; DOI: 10.2172/4018518.

(S5) Haley, F. A. *Effects of Mixed Field Radiation on Lubricating Oils*; 1961.

(S6) Beerbower, A.; Murray, J. L. Radiation Resistant Mineral Oils, US Patent 3094488. 1963.

(S7) Mahoney, C. L.; Barnum, E. R.; Kerlin, W. W.; Sax, K. J.; Saari, W. S. Polyphenyl Ethers as High-Temperature Radiation-Resistant Lubricants. *J. Chem. Eng. Data* **1960**, *5*, 172–180, DOI: 10.1021/je60006a013.

(S8) van de Voorde, M. *Radiation Resistance of Lubricants, CERN-ISR-MAG-68-14*; 1968.

(S9) Coleman, T. L. *Nuclear and Space Radiation Effects on Materials - Space Vehicle Design Criteria*; 1970.

(S10) Bouquet, F. L.; Newell, D. M.; Price, W. E. Designer's Guide to Radiation Effects on Materials for Use on Jupiter Fly-Bys and Orbiters. *IEEE Trans. Nucl. Sci.* **1979**, *26*, 4660–4669, DOI: 10.1109/TNS.1979.4330186.

(S11) Potanina, V. A.; Zherdeva, L. G.; Gorbatch, V. A.; Siryuk, A. G.; Zaslavskii, Y. S.; Ponomareva, T. P.; Smirnyagina, N. A. Effect of Ionizing Radiation on Naphthenic Hydrocarbon in Lubricating Oils. *Chem. Technol. Fuels Oils* **1976**, *10*, 14–18, DOI: 10.1007/bf00718759.

(S12) Potanina, V. A.; Gorbatch, V. A.; Siryuk, A. G.; Zaslavskii, Y. S.; Ponomareva, T. P. Effect of Ionizing Radiation on Aromatic Hydrocarbons in Lubricating Oils. *Chem. Technol. Fuels Oils* **1977**, *8*, 25–28, DOI: 10.1007/bf00719086.

(S13) Fadel, M. A.; Aboul-Gheit, A. K.; Al-Alousi, D. S. Radiation Effects on a Lubricating Base Oil and Their Application in Radiation Dosimetry β. *Radiat. Eff.* **1979**, *41*, 245–250, DOI: 10.1080/00337577908236973.

(S14) Bruce, M. B.; Davis, M. V. *Radiation Effects on Organic Materials in Nuclear Plants. Final Report*; 1981; DOI: 10.2172/5591289.

(S15) International Atomic Energy Agency *Radiation Damage to Organic Materials in Nuclear Reactors and Radiation Environments*; 1990; pp 1–164.

(S16) Schönbacher, H.; Tavlet, M. *Radiation Effects on Structural Materials for High-Energy Particle Accelerators and Detectors*; 1994.

(S17) Nakanishi, H.; Arakawa, K.; Hayakawa, N.; Machi, S.; Yagi, T. *Radiation Effect of Aromatic Lubricating Oils*; 1984.

(S18) Arakawa, K.; Hayakawa, N.; Yoshida, K.; Tamura, N.; Nakanishi, H.; Yagi, T.; Kuroiwa, S. Lubricating Oil Blend Resistant to Ionizing Radiation, US Patent 4664829. 1987.

(S19) Arakawa, K.; Nakanishi, H.; Morishita, N.; Soda, T.; Hayakawa, N.; Yagi, T.; Machi, S. *Data on Radiation Resistance of Lubricating Oil*; 1987.

(S20) Shul'zhenko, I. V.; Kobzova, R. I.; Chepurova, M. B.; Izotov, Y. P. Effect of Low-Power Ionizing Radiation on Properties of Lubricating Greases. *Chem. Technol. Fuels Oils* **1981**, *17*, 217–219, DOI: 10.1007/bf00730590.

(S21) Arakawa, K.; Hayakawa, N.; Yoshida, K.; Yagi, T.; Nakanishi, H. Radiation-Resistant Grease, US Patent 4711732. 1987.

(S22) Arakawa, K.; Sasuga, T.; Hagiwara, M.; Hayakawa, N.; Yoshida, K.; Nakanishi, H.; Hirohama, M.; Yagi, T.; Akada, T.; Soda, T. Super Highly Radiation-Resistant Grease, US Patent 4753741. 1988.

(S23) Ferrari, M.; Zenoni, A.; Hartl, M.; Lee, Y.; Andrighetto, A.; Monetti, A.; Salvini, A.; Zelaschi, F. Experimental Study of Consistency Degradation of Different Greases in Mixed Neutron and Gamma Radiation. *Heliyon* **2019**, *5*, e02489, DOI: 10.1016/j.heliyon.2019.e02489.

(S24) Ferrari, M.; Senajova, D.; Kershaw, K.; Perillo Marcone, A.; Calviani, M. Selection of Radiation Tolerant Commercial Greases for High-Radiation Areas at CERN: Methodology and Applications. *Nucl. Mater. Energy* **2021**, *29*, 101088, DOI: 10.1016/j.nme.2021.101088.

(S25) Lv, M.; Wang, H.; Wang, L.; Kong, F.; Wang, T.; Wang, Q. The Effect of Space Irradiation on the Lubricating Performance of Perfluoropolyether Greases in Simulated Space Environment. *Lubr. Sci.* **2017**, *29*, 567–575, DOI: 10.1002/ls.1390.

(S26) Obara, K.; Kakuuate, S.; Oka, K.; Taguchi, H.; Ito, A.; Koizumi, K.; Shibanuma, K.; Yagi, T.; Morita, Y.; Kanazawa, T.; Tada, E. *High Gamma-Rays Irradiation Tests of Critical Components for ITER (International Thermonuclear Experimental Reactor) In-Vessel Remote Handling System*; 1999.

(S27) Onishi, Y.; Shiga, T.; Ohkawa, Y.; Katoh, H.; Nagasawa, K.; Okada, T.; Saimen, K.; Itano, F.; Murano, Y.; Tsujita, Y.; Yagi, T.; Morita, Y.; Seguchi, T. Study on Polymer Materials for Development of the Super 100 MGy-Radiation Resistant Motor. *Polym. J.* **2004**, *36*, 617–622, DOI: 10.1295/polymj.36.617.

(S28) Onishi, Y.; Shiga, T.; Ohkawa, Y.; Katoh, H.; Nagasawa, K.; Okada, T.; Saimen, K.; Itano, F.; Murano, Y.; Tsujita, Y.; Yagi, T.; Morita, Y.; Seguchi, T. Development of Super 100-MGy Radiation-Durable Motor and Study of Radiation Resistance Mechanism. *IEEE Trans. Energy Convers.* **2005**, *20*, 693–699, DOI: 10.1109/TEC.2005.850271.

(S29) Nakamichi, M.; Ishitsuka, E.; Shimakawa, S.; Kan, S. Irradiation Tests of a Small-Sized Motor with Radiation Resistance. *Fusion Eng. Des.* **2008**, *83*, 1321–1325, DOI: 10.1016/J.FUSENGDES.2008.08.033.

(S30) Saito, M.; Anzai, K.; Maruyama, T.; Noguchi, Y.; Ueno, K.; Takeda, N.; Kakudate, S. Development of Radiation Hard Components for ITER Blanket Remote Handling

System. *Fusion Eng. Des.* **2016**, *109-111*, 1502–1506, DOI: 10.1016/j.fusengdes.2015.11.042.

(S31) Bliznyuk, V. N.; Smith, J.; Guin, T.; Verst, C.; Folkert, J.; McDonald, K.; Larsen, G.; DeVol, T. A. Photoluminescence Induced in Mineral Oil by Ionizing Radiation. *Lubricants* **2023**, *11*, 287, DOI: 10.3390/LUBRICANTS11070287.

(S32) Ferrari, M. Experimental Study of Radiation Resistance in Intense Neutron Fields of Critical Materials and Components for the Construction of the ESS (European Spallation Source) Target System. Ph.D. thesis, Università degli Studi di Brescia, 2020.

(S33) Maestre, J.; Torregrosa, C.; Kershaw, K.; Bracco, C.; Coiffet, T.; Ferrari, M.; Franqueira Ximenes, R.; Gilardoni, S.; Grenier, D.; Lechner, A.; Maire, V.; Martin Ruiz, J. M.; Matheson, E.; Solieri, N.; Perillo-Marcone, A.; Polzin, T.; Rizzoglio, V.; Senajova, D.; Sharp, C.; Timmins, M.; Calviani, M. Design and Behaviour of the Large Hadron Collider External Beam Dumps Capable of Receiving 539 MJ/Dump. *J. Instrum.* **2021**, *16*, P11019, DOI: 10.1088/1748-0221/16/11/P11019.

(S34) Ferrari, M.; Zenoni, A.; Lee, Y. J.; Andrighetto, A. Neutron Radiation Effects on Lubricants and O-Rings for Target and Accelerator Applications. *Mater. Sci. Forum* **2021**, *1024*, 127–133, DOI: 10.4028/www.scientific.net/MSF.1024.127.

(S35) Carbajo Perez, D.; Perillo-Marcone, A.; Berthome, E.; Bertone, C.; Biancacci, N.; Bracco, C.; Bregliozzi, G.; Bulat, B.; Cadiou, C.; Calviani, M.; Cattenoz, G.; Cherif, A.; Costa Pinto, P.; Dallocchio, A.; Di Castro, M.; Fessia, P.; Frankl, M. I.; Fuchs, J. F.; Garcia Gavela, H.; Gentini, L.; Geisser, J. M.; Gilardoni, S.; Gonzalez De La Aleja Cabana, M. A.; Grenard, J. L.; Joly, S.; Lechner, A.; Lendaro, J.; Maestre, J.; Page, E.; Perez Ornedo, M.; Pugnat, D.; Rigutto, E.; Seidenbinder, R.; Salvant, B.; Sapountzis, A.; Scibor, K.; Sola Merino, J.; Taborelli, M.; Urrutia, E.; Franqueira Ximenes, R.; Vieille, A.; Vollinger, C.; Yin Vallgren CERN, C. New Gener-

ation CERN LHC Injection Dump - Assembly and Installation (TDIS). *JaCOW IPAC* **2021**, DOI: 10.18429/JACoW-IPAC2021-WEPAB361.

(S36) Ferrari, M.; Alia, R. G.; Giles, T.; Senajova, D.; Pandini, S.; Zenoni, A.; Calviani, M. "Radiation to Materials" at CERN. *IEEE Trans. Nucl. Sci.* **2023**, *70*, 1580–1586, DOI: 10.1109/TNS.2023.3241785.

(S37) Lilli, G.; Centofante, L.; Manzolaro, M.; Monetti, A.; Oboe, R.; Andrighetto, A. Remote Handling Systems for the Selective Production of Exotic Species (SPES) Facility. *Nucl. Eng. Technol.* **2023**, *55*, 378–390, DOI: 10.1016/J.NET.2022.08.034.

(S38) Khan, M. A.; Hopkins, D. P. Radiation Compatible Lubricant for Medical Devices, US Patent 6102898. 2000.

(S39) Hayashi, Y. The World's Highest Radiation-Resistant Lubricant Supporting Decommissioning of the Nuclear Reactor. 2022; Abstracts of the Technical Poster Session, 1FD6, The 6th International Forum on the Decommissioning of the Fukushima Daiichi Nuclear Power Station, Alios Iwaki Performing Art Center, Fukushima Prefecture, Japan.

(S40) Singh, A.; Verma, N.; Mamatha, T. G.; Kumar, A.; Singh, S.; Kumar, K. Properties, Functions and Applications of Commonly Used Lubricant Additives: A Review. *Mater. Today Proc.* **2021**, *44*, 5018–5022, DOI: 10.1016/J.MATPR.2021.01.029.

(S41) Mortier, R. M.; Fox, M. F.; Orszulik, S. T. *Chemistry and Technology of Lubricants*, 3rd ed.; Springer, 2010.

(S42) Rudnick, L. R. *Synthetics, Mineral Oils and Bio-Based Lubricants: Chemistry and Technology*; CRC Press, 2006.

(S43) Lugt, P. M. *Grease Lubrication in Rolling Bearings*; John Wiley & Sons, Ltd., 2013; DOI: 10.1002/9781118483961.

(S44) Gurt, A.; Khonsari, M. M. Testing Grease Consistency. *Lubricants* **2021**, *9*, 14, DOI: 10.3390/lubricants9020014.

(S45) Wang, Y.; Zhang, P.; Lin, J.; Gao, X. Rheological and Tribological Properties of Lithium Grease and Polyurea Grease with Different Consistencies. *Coatings* **2022**, *12*, 527, DOI: 10.3390/coatings12040527.

(S46) Tomaru, M.; Suzuki, T.; Ito, H.; Suzuki, T. Grease-Life Estimation and Grease Deterioration in Sealed Ball Bearings. Proceedings of the JSLE International Tribology Conference. 1985.

(S47) Stodola, J.; Stodola, P. Controlled Degradation of Lubricating Media by Means of an Accelerated Electron Beam. *Processes* **2020**, *8*, 1452, DOI: 10.3390/PR8111452.

(S48) Ishitsuka, E.; Kan, S.; Kawamura, H.; Onozawa, H. In Situ Characterization of a Small Sized Motor under Neutron Irradiation. *Fusion Eng. Des.* **2001**, *58–59*, 517–521, DOI: 10.1016/S0920-3796(01)00241-1.

(S49) Ferrari, M.; Zenoni, A.; Lee, Y.; Hayashi, Y. Characterization of a Polyphenyl Ether Oil Irradiated at High Doses in a Triga Mark II Nuclear Reactor. *Nucl. Instrum. Methods Phys. Res. B* **2021**, *497*, 1–9, DOI: 10.1016/j.nimb.2021.03.021.

(S50) Knowles, E. C.; Lyons, J. F.; Odell, N. R. Radiation Resistant Lubricating Grease, US Patent 3288711. 1966.

(S51) Obara, K.; Kakudate, S.; Oka, K.; Furuya, K.; Taguchi, H.; Tada, E.; Shibanuma, K.; Koizumi, K.; Ohkawa, Y.; Morita, Y.; Yagi, T.; Yokoo, N.; Kanazawa, T.; Haneda, N.; Kaneko, H. *Irradiation Tests of Critical Components for Remote Handling System in Gamma Radiation Environment*; 1996.

(S52) Cheng, X. Nanostructures: Fabrication and Applications. In *Nanolithography: The Art*

of Fabricating Nanoelectronic and Nanophotonic Devices and Systems; Feldman, M., Ed.; Woodhead Publishing, 2014; pp 348–375, DOI: 10.1533/9780857098757.348.

(S53) Rahman, M. M.; Islam, M.; Roy, R.; Younis, H.; AlNahyan, M.; Younes, H. Carbon Nanomaterial-Based Lubricants: Review of Recent Developments. *Lubricants* **2022**, *10*, 281, DOI: 10.3390/lubricants10110281.

(S54) Sun, J.; Du, S. Application of Graphene Derivatives and Their Nanocomposites in Tribology and Lubrication: A Review. *RSC Adv.* **2019**, *9*, 40642–40661, DOI: 10.1039/C9RA05679C.

(S55) Ali, I.; Basheer, A. A.; Kucherova, A.; Memetov, N.; Pasko, T.; Ovchinnikov, K.; Pershin, V.; Kuznetsov, D.; Galunin, E.; Grachev, V.; Tkachev, A. Advances in Carbon Nanomaterials as Lubricants Modifiers. *J. Mol. Liq.* **2019**, *279*, 251–266, DOI: 10.1016/j.molliq.2019.01.113.

(S56) Xu, H.; Akbari, M. K.; Zhuiykov, S. 2D Semiconductor Nanomaterials and Heterostructures: Controlled Synthesis and Functional Applications. *Nanoscale Res. Lett.* **2021**, *16*, 94, DOI: 10.1186/S11671-021-03551-W.

(S57) Nyholm, N.; Espallargas, N. Functionalized Carbon Nanostructures as Lubricant Additives – A Review. *Carbon* **2023**, *201*, 1200–1228, DOI: 10.1016/j.carbon.2022.10.035.

(S58) Liu, L.; Zhou, M.; Jin, L.; Li, L.; Mo, Y.; Su, G.; Li, X.; Zhu, H.; Tian, Y. Recent Advances in Friction and Lubrication of Graphene and Other 2D Materials: Mechanisms and Applications. *Friction* **2019**, *7*, 199–216, DOI: 10.1007/s40544-019-0268-4.

(S59) Xiao, H.; Liu, S. 2D Nanomaterials As Lubricant Additive: A Review. *Mater. Design* **2017**, *135*, 319–332, DOI: 10.1016/j.matdes.2017.09.029.

(S60) Zin, V.; Agresti, F.; Barison, S.; Colla, L.; Mercadelli, E.; Fabrizio, M.; Pagura, C. Tribological Properties of Engine Oil with Carbon Nano-Horns As Nano-Additives. *Tribol. Lett.* **2014**, *55*, 45–53, DOI: 10.1007/s11249-014-0330-3.

(S61) Tao, X.; Jiazheng, Z.; Kang, X. The Ball-Bearing Effect of Diamond Nanoparticles As an Oil Additive. *J. Phys. D Appl. Phys.* **1996**, *29*, 2932–2937, DOI: 10.1088/0022-3727/29/11/029.

(S62) Marian, M.; Feile, K.; Rothammer, B.; Bartz, M.; Wartzack, S.; Seynstahl, A.; Tremmel, S.; Krauß, S.; Merle, B.; Böhm, T.; Wang, B.; Wyatt, B. C.; Anasori, B.; Rosenkranz, A. $Ti_3C_2T_x$ Solid Lubricant Coatings in Rolling Bearings with Remarkable Performance beyond State-of-the-Art Materials. *Appl. Mater. Today* **2021**, *25*, DOI: 10.1016/j.apmt.2021.101202.

(S63) Berman, D.; Erdemir, A.; Sumant, A. V. Graphene: A New Emerging Lubricant. *Mater. Today* **2014**, *17*, 31–42, DOI: 10.1016/j.mattod.2013.12.003.

(S64) Yin, X.; Wu, F.; Chen, X.; Xu, J.; Wu, P.; Li, J.; Zhang, C.; Luo, J. Graphene-Induced Reconstruction of the Sliding Interface Assisting the Improved Lubricity of Various Tribo-Couples. *Mater. Design* **2020**, *191*, 108661, DOI: 10.1016/J.MATDES.2020.108661.

(S65) Kogovšek, J.; Kalin, M. Lubrication Performance of Graphene-Containing Oil on Steel and DLC-Coated Surfaces. *Tribol. Int.* **2019**, *138*, 59–67, DOI: 10.1016/J.TRIBOINT.2019.05.026.

(S66) Joly-Pottuz, L.; Vacher, B.; Ohmae, N.; Martin, J. M.; Epicier, T. Anti-Wear and Friction Reducing Mechanisms of Carbon Nano-Onions As Lubricant Additives. *Tribol. Lett.* **2008**, *30*, 69–80, DOI: 10.1007/s11249-008-9316-3.

(S67) Grützmacher, P. G.; Suarez, S.; Tolosa, A.; Gachot, C.; Song, G.; Wang, B.; Presser, V.;

Mücklich, F.; Anasori, B.; Rosenkranz, A. Superior Wear-Resistance of $Ti_3C_2T_x$ Multilayer Coatings. *ACS Nano* **2021**, *15*, 8216–8224, DOI: 10.1021/acsnano.1c01555.

(S68) Shang, W.; Ye, M.; Cai, T.; Zhao, L.; Zhang, Y.; Liu, D.; Liu, S. Tuning of the Hydrophilicity and Hydrophobicity of Nitrogen Doped Carbon Dots: A Facile Approach Towards High Efficient Lubricant Nanoadditives. *J. Mol. Liq.* **2018**, *266*, 65–74, DOI: 10.1016/j.molliq.2018.06.042.

(S69) Gulzar, M.; Masjuki, H. H.; Kalam, M. A.; Varman, M.; Zulkifli, N. W. M.; Mufti, R. A.; Zahid, R. Tribological Performance of Nanoparticles As Lubricating Oil Additives. *J. Nanopart. Res.* **2016**, *18*, 1–25, DOI: 10.1007/S11051-016-3537-4.

(S70) Nunn, N.; Mahbooba, Z.; Ivanov, M. G.; Ivanov, D. M.; Brenner, D. W.; Shenderova, O. Tribological Properties of Polyalphaolefin Oil Modified with Nanocarbon Additives. *Diam. Relat. Mater.* **2015**, *54*, 97–102, DOI: 10.1016/j.diamond.2014.09.003.

(S71) Marchetto, D.; Restuccia, P.; Ballestrazzi, A.; Righi, M. C.; Rota, A.; Valeri, S. Surface Passivation by Graphene in the Lubrication of Iron: A Comparison with Bronze. *Carbon* **2017**, *116*, 375–380, DOI: 10.1016/j.carbon.2017.02.011.

(S72) Restuccia, P.; Righi, M. C. Tribochemistry of Graphene on Iron and Its Possible Role in Lubrication of Steel. *Carbon* **2016**, *106*, 118–124, DOI: 10.1016/j.carbon.2016.05.025.

(S73) Spikes, H. Friction Modifier Additives. *Tribol. Lett.* **2015**, *60*, 1–26, DOI: 10.1007/S11249-015-0589-Z.
